# Extended genotypic evaluation and comparison of twenty-two cases of lethal EEHV1 hemorrhagic disease in wild and captive Asian elephants in India

**DOI:** 10.1371/journal.pone.0202438

**Published:** 2018-08-22

**Authors:** A. Zachariah, P. K. Sajesh, S. Santhosh, C. Bathrachalam, M. Megha, J. Pandiyan, M. Jishnu, R. S. Kobragade, S. Y. Long, J-C Zong, E. M. Latimer, S. Y. Heaggans, G. S. Hayward

**Affiliations:** 1 Department of Forests and Wildlife, Government of Kerala, Sultan Battery, Wayanad, India; 2 SciGenom Research Foundation, Cheruthuruthy, Kerala, India; 3 AVC College, Mayiladuthurai, Tamilnadu, India; 4 Tadoba-Andhari Tiger Reserve, Chandrapur, Maharashtra, India; 5 Viral Oncology Program, Department of Oncology, Johns Hopkins School of Medicine, Baltimore, MD, United States of America; 6 Wildlife Health Sciences, Smithsonian’s National Zoological Park, Washington, DC, United States of America; Banaras Hindu University, INDIA

## Abstract

Thirteen new lethal cases of acute hemorrhagic disease (HD) with typical histopathogical features were identified in young Asian elephants (*Elephas maximus indicus)* in India between 2013 and 2017. Eight occurred amongst free-ranging wild herds, with three more in camp-raised orphans and two in captive-born calves. All were confirmed to have high levels of Elephant Endotheliotropic Herpesvirus type 1A (EEHV1A) DNA detected within gross pathological lesions from necropsy tissue by multi-locus PCR DNA sequencing. The strains involved were all significantly different from one another and from nine previously described cases from Southern India (which included one example of EEHV1B). Overall, eight selected dispersed PCR loci totaling up to 6.1-kb in size were analyzed for most of the 22 cases, with extensive subtype clustering data being obtained at four hypervariable gene loci. In addition to the previously identified U48(gH-TK) and U51(vGPCR1) gene loci, these included two newly identified E5(vGPCR5) and E54(vOX2-1) loci mapping far outside of the classic EEHV1A versus EEHV1B subtype chimeric domains and towards the novel end segments of the genome that had not been evaluated previously. The high levels of genetic divergence and mosaic scrambling observed between adjacent loci match closely to the overall range of divergence found within 45 analyzed North American and European cases, but include some common relatively unique polymorphic features and preferred subtypes that appear to distinguish most but not all Indian strains from both those in Thailand and those outside range countries. Furthermore, more than half of the Indian cases studied here involved calves living within wild herds, whereas nearly all other cases identified in Asia so far represent rescued camp orphans or captive-born calves.

## Introduction

The deaths of more than 100 young endangered Asian elephant calves worldwide with acute hemorrhagic disease (HD) have been documented to be associated with a novel type of herpesvirus called Elephant Endotheliotropic herpesvirus (EEHV) found at very high levels in blood or necropsy tissue samples. The first reported HD case occurred in a calf in a circus in Switzerland [1] and the discovery of DNA from the causative agent EEHV1 in a 16-month old female calf at the National Zoological Park in Washington DC in 1995, as well as in ten other archival cases, was described by Richman et al [2, 3]. The victims have primarily been between one and eight years of age with a peak between 2 and 4 years and have included the first Asian calves born at the Bronx and National Zoos, as well as the first Asian calves in both the USA and Europe conceived by AI techniques [4–6]. The disease proceeds rapidly over just a few days with symptoms of lethargy, edema and tongue cyanosis, followed by damaged vascular endothelial cells and hemorrhaging in all major organs, culminating in very high levels of viral DNA in peripheral mononuclear white blood cells and serum and with a near total loss of platelets or thrombocytopenia [7]. About a dozen seriously ill calves treated with human anti-herpesvirus drugs such as ACV, FCV and GCV are known to have survived [8], whereas many others did not, but the available evidence suggests that early detection resulting in rapid supportive medical care and treatment of the symptoms is likely more effective than the drugs themselves [5, 7].

Over the past 20 years, acute hemorrhagic disease occurring in young Asian elephants (*Elephas maximus*) born in captivity has been observed to have a fatality rate of about 80%. Overall, as reviewed by Long et al [5], EEHV HD has affected more than 20% of all Asian elephant calves born in North American and European zoos between 1995 and 2015, and has been the cause of 65% of the deaths that occurred amongst North American zoo or circus reared calves during that time period. Both lethal and symptomatic surviving cases proved to have massive systemic viremia with one of up to seven known species of EEHV. These presumed primary infections produce lytic infection in vascular endothelial cells with extremely high viral DNA loads in blood and affected internal organs. Many healthy Asian and African adult elephants carry and occasionally shed one of several distinctive species of EEHV each in saliva and trunk washes, but evidently only EEHV1A has unusually high pathogenicity accounting for more than 90% of symptomatic cases. Although a similar lethal disease had been rumored to occur in Asian range countries in the mid-1990s, this was only confirmed also to be EEHV HD much more recently by conventional PCR DNA sequencing of multiple cases mostly in India and Thailand.

Although this disease predominantly affects Asian elephants, a second related virus EEHV2 was also found by Richman et al [2] to be present in a rare but similar lethal case in a young African elephant. Since then, five additional EEHV species have been identified in other Asian and African elephants with mild or lethal acute systemic disease [9–14] and in lung nodules, as well as at low levels in some random necropsy tissue samples from healthy adult African elephants [15]. Specific DNA-based PCR blood and trunk wash tests that were developed to detect all seven known EEHV species [10, 15–18] have revealed that these viruses are natural and largely benign infections endemic to both Asian and African elephants in the wild, as well as in zoos. However, primary infection with predominantly just the one highly pathogenic type EEHV1A leads to lethal HD in up to 80% of Asian calves that develop characteristic acute clinical symptoms, although many others survive with lower grade or even asymptomatic mild disease [4, 17, 19]. Overall, as reviewed by Long et al [5], EEHV HD has affected more than 20% of all Asian elephant calves born in North American and European zoos between 1995 and 2015, and has been the cause of 65% of the deaths that occurred amongst North American zoo or circus reared calves during that time period.

In our initial collaborative studies in Asia, EEHV1-specific DNA PCR assays were used to confirm nine similar cases of lethal EEHV HD among camp or zoo-reared orphans, as well as several free-ranging wild calves in Southern India [20]. Others have also reported lethal DNA-confirmed cases particularly from Thailand, but also several in Cambodia and Laos [21–24]. All cases identified so far in Asian elephants in range countries have involved predominantly EEHV1A or occasionally EEHV1B or EEHV4.

The intact DNA genomes of each of the four major types of EEHV known to cause HD in Asian elephant calves (EEHV1A, EEHV1B, EEHV4 and EEHV5) have now all been completely sequenced directly from necropsy tissue samples [25–30]. In contrast to the virus species found in Asian elephants, four other EEHV species namely EEHV2, EEHV3, EEHV6 and EEHV7, have been detected in African elephants [10, 15]. These viruses from both elephant host species fall into a single but highly diverged new clade of mammalian herpesviruses known as the *Proboscivirus* genus that is distinctly different from any of the genera in each of the three currently assigned subfamilies [13, 14, 29–31]. Furthermore, they form two major branches of AT-rich or GC-rich genomes between 180 to 206-kb in size and encoding approximately 118 genes, half of which are novel to the *Proboscivirus* genus. Each species of EEHV also divides into at least two major chimeric subtypes of which EEHV1A and EEHV1B are evidently the most prevalent and pathogenic virus types. DNA sequence characterization of the EEHV1 genomes from all 35 known lethal and surviving HD cases in North America, as well as ten more from Europe, have revealed that a selective subset of gene loci within non-epidemiologically related EEHV1 strains display high levels of strain-specific nucleotide and amino acid variability. This data permits each individual virus strain encountered to be assigned to one of multiple very distinctive subtype clusters at each of the variable gene loci [15].

Our earlier report involving DNA sequence data from several selected PCR gene loci from the first nine cases from India [20] revealed that in addition to there being eight examples of EEHV1A and one of EEHV1B, two cases occurring in unrelated calves just a few days apart at the same camp facility involved identical EEHV1A strains (IP06 and IP07), whereas the other six EEHV1As displayed almost as much strain variability as found across all 45 cases previously studied from European and North American zoos. Those results clearly support the concept that EEHV cases amongst Asian elephants under human care in Western countries derive from viruses carried latently within earlier generations of wild elephants imported from Asian range countries. Furthermore, based on the very large number of distinct strains of EEHV1 detected and the observation that no two facilities in the USA or elsewhere have the same strains, whereas many facilities have multiple species and strains present, the disease clearly has a sporadic not epidemic nor zoonotic pattern [4, 5]. Recent studies on Thailand HD cases have detected several more examples of EEHV1B and EEHV4 there in addition to 13 examples of the more common EEHV1A subtype [21, 23]. Analysis of the nine initial EEHV1 cases from India included PCR DNA sequence data from up to six selected gene loci (totaling 3,300-bp) across the conserved central core segments of the EEHV1 genome, including two hypervariable loci representing the U48(gH-TK) and U51(vGPCR1) gene regions that each fall into five or six clustered but unlinked subtypes.

**Fig 1:**
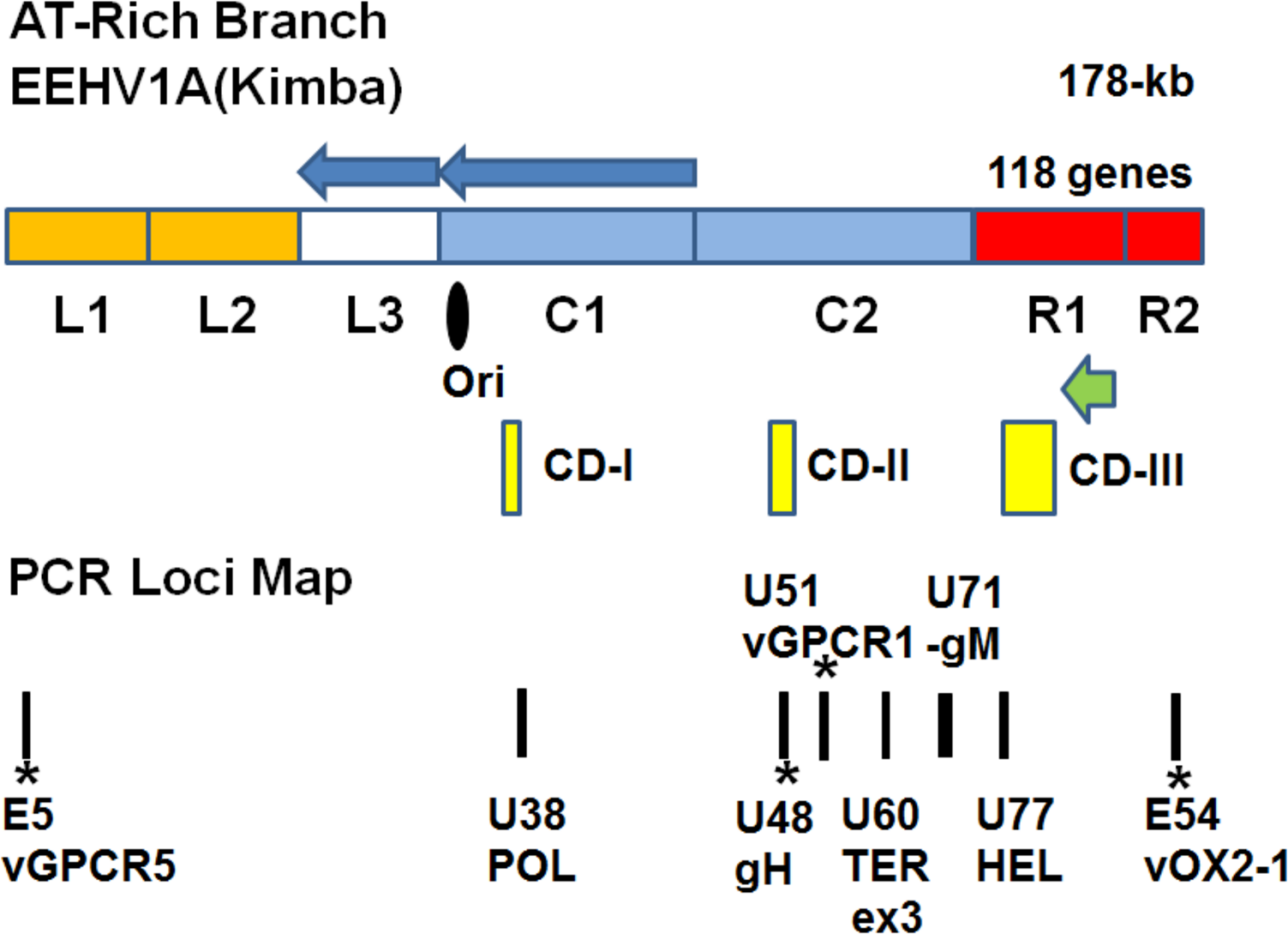
Genome map location of the eight sequenced PCR loci involved in these studies. The diagram shows the relative to-scale positions of seven regions within the nearly 180-kb EEHV1A(Kimba) genome encompassing both conserved core (C1 and C2) and novel (L1, L2, L3, R1, R2) segments as defined in Ling et al [30]. The map includes denoting positions of the predicted Ori-Lyt domain (black oval) and the likely major transcriptional regulatory protein genes (ORF-K and ORF-L, leftwards green arrow). The three major chimeric domains (CD-I, CD-II, CD-III) are shown as yellow boxes and the overall inverted orientation of segments L3 and C1 relative to other herpesvirus subfamilies are indicated by the blue arrows. The eight amplified PCR loci involved in these studies (black bars) total 6.5-kb in size, with four of them representing non-CD core segments (3% diverged between subtype A and B) whereas the four with asterisks encompass hypervariable loci of which the two centrally located ones U48(gH) and U51(vGPCR1) still display classic A/B subtype patterns, whereas the other two E5(vGPCR5) and E54(vOX2-1) located within the outer unique regions towards the ends of the genome do not.

The present expanded genetic analyses reveal that there are many different ancient and highly diverged variants of EEHV1A associated with lethal HD disease in Southern India. Overall, proven or anecdotal cases are widespread in Asia and have now been reported in at least eight different range countries, indicating that EEHV HD represents a serious additional threat to the current and future reproductive success in zoos and the conservation of highly endangered free-ranging wild populations worldwide. In the new studies reported here, we have also examined a further 12 EEHV1-positive lethal cases associated with the two largest elephant sub-populations from the Western Gnats area of Southern India plus one case from Central India. In addition to the same six original PCR loci, two other variable PCR gene loci from the ends of the EEHV1 genome referred to as E54(vOX2-1) and E5(vGPCR5) [25] were included, together with an expanded longer version of the U60(TERex3) locus for both the new and several of the earlier India cases (**Fig 1**). The novel hypervariable E54(OX2-1) gene data from the unique segment shows no linkage at all to the standard EEHV1A versus EEHV1B chimerism patterns displayed by all six of the core gene loci examined previously [14], and is not readily categorized into subtypes. However, it does display up to 15% overall nucleotide level variability and has an advantage over the other PCR loci in distinguishing unambiguously between most known EEHV1 strains, including those within the usually highly conserved EEHV1B subset. Similarly, the new E5(vGPCR1) locus also shows no linkage to the standard EEHV1A versus EEHV1B chimeric patterns either, but instead splits into three distinct groups referred to as A, B and C. When all eight PCR loci are included the total combined DNA sequences analyzed totals close to 6,100-bp. The results obtained were also compared to matching data for five more fatal Asian range country cases that have been evaluated recently, including two identical EEHV1A cases from Sumatra and three cases involving distinct EEHV1A strains from Myanmar. Those latter results will be reported on in greater detail elsewhere (Stremme C. et al, submitted; Oo Z.M. et al, submitted).

## Results

### Identification and pathology of positive case

Twelve of the new cases of sudden HD deaths in young Asian elephants in Southern India were collected from a variety of locations across the Western Gnats area encompassing parts of Kerala State, whereas one came from Maharishtra State outside of this area. A list summarizing the major features and details available about each of the 13 new HD cases is provided in **Table 1**. They all displayed gross pathological features typical of those described previously [3, 20], including facial and neck edema, as well as internal hemorrhaging and petechiae in most major internal organs. In some cases, typical histopathologically identifiable herpesvirus-like intranuclear inclusion bodies were also observed within vascular endothelial cells. Seven of these calves were found post-mortem by the field staff amongst wild free-ranging herds, whereas three others occurred amongst wild-born orphans in camps or in otherwise closely monitored human care situations and two were captive-born. Necropsies were performed as soon as possible and tissue samples (usually from heart, liver or lung) were stored initially in 100% ethanol then later frozen at -80°C before DNA extraction. In the initial diagnostic testing, PCR with pan-EEHV POL primers was carried out on DNA samples extracted from at least one necropsy tissue sample for each new case. These samples all gave first round positive bands after agarose gel electrophoresis, that migrated at the expected 480-bp size for this locus when amplified from EEHV genomic DNA. Subsequent standard Sanger cycle DNA sequencing analyses then revealed typical EEHV1A profiles rather than the other plausible options for Asian elephants of EEHV1B, EEHV4 or EEHV5.

**Table 1:** Detailed descriptions of the new Indian cases.

Cases 1 to 9: Details of the nine Indian cases evaluated from between 2005 and 2011 were provided previously in Table 1 of Zachariah et al [20]. Four involved wild free-ranging elephants (IP05, IP11, IP60 and IP91), two were in rescued orphans housed in the same training camp at Kodanad (IP06, IP07) and another orphan was being housed at the Arigna Anna Zoo in Chennai (IP43), whereas two (IP01 and IP93) were in captive-born elephants, with the latter having originated in the Andaman Islands). Three of the wild-born cases (IP11, IP43 and IP91) occurred within areas north of the Palaghat gap (Nilgiri Biosphere Reserve = NBR) and four (IP05, IP06, IP07 and IP60) occurred south of the Palaghat gap (Anamalai/Periyar region = A/P). Note that IP06 (Nilambur) and IP07 (Munnar) were orphaned in different areas within the Western Knats.
Case 10: Kozhikode1 (IP143, A/P). Eight-year old captive born male. Died Apr 26^th^ 2013 with typical gross lesions in internal organs and enlarged temporal lobe. Histopathology positive for tissue hemorrhaging and intranuclear inclusion bodies.
Case 11: Nilambur (IP152, NBR). Two-year old wild free-ranging female calf. Died with gross hemorrhagic lesions on Aug 27^th^ 2013.
Case 12: Muthanga (IP164, NBR). A 16 month old, wild-born orphan raised in a camp. Died in 2014 with typical gross hemorrhagic lesions. Histopathology positive for tissue hemorrhaging and intranuclear inclusion bodies.
Case 13: Thirunelli2 (IP165, NBR). A two-year-old wild free-ranging male that was abandoned by its herd. Died suddenly on Feb 9^th^ 2014 with typical gross lesions. Histopathology positive for hemorrhaging and intranuclear inclusion bodies.
Case 14: Kazhargod (IP169, NBR). Seven-year-old wild free-ranging male. Died suddenly with gross hemorrhagic lesions on Oct 20^th^ 2014.
Case 15: Kozhikode2 (IP183, NBR). Two-year-old, wild free-ranging female. Died Mar 12^th^ 2016 in Thamrassery range, with typical gross hemorrhagic lesions. Histopathology positive for inclusion bodies.
Case 16: Konni1 (IP200). Five-year-old wild-born orphan female managed in captivity at Konni Camp, Pathanamthitta District (A/P). Died suddenly in July 2016 with multiorgan gross lesions. Originally rescued as a one-year-old from Malampuzha in the Palakhad district (NPR). Histopathology positive for intranuclear inclusion bodies.
Case 17: Munnar (IP208, A/P). Four-year-old wild free-ranging male. Died Sep 11^th^ 2016 with gross multi-organ hemorrhagic lesions.
Case 18: Konni2 (IP212). An eighteen-month-old female orphan. Died suddenly May 12^th^ 2016 in Konni Camp (A/P). Originally rescued at eight months old from Karulai forest, Nilambur (NBR), Kerala. Multi-organ hemorrhages, with intranuclear inclusion bodies in the heart.
Case 19: Tadoba (IP213, CR). Captive-born 14-month-old male calf. Died May 2cd 2016 in Tadoba-Andhari Tiger Reserve, Maharashtra (Central region), with typical gross hemorrhagic lesions.
Case 20: Periyar1 (IP222, A/P). A wild-born free ranging male calf less than one-year-old. Died in Mar 2017 in Periyar Tiger Reserve, with typical gross hemorrhagic lesions.
Case 21: Tholpetty (IP239, NBR). A wild-born free ranging two-year-old male calf. Died in the Tholpetty range within the Weyanad Wildlife Sanctuary on May 27 2017. Typical gross hemorrhagic lesions.
Case 22: Periyar2 (IP240, A/P). A wild-born free ranging two-year old female calf from the Vandakadvu range in the Periyar Tiger reserve. Necropsy examination on Jun 18^th^ 2017 revealed typical gross hemorrhagic pathology.

### Gene subtype DNA sequence analysis

In our previous studies worldwide, four separate PCR loci mapping across the central core segment of the EEHV1 genomes have been found to give consistent diagnostic distinctions between the two partially chimeric EEHV1A or EEHV1B genotypes, in which all examples within each of the two groups show common patterns of approximately 3.5% nucleotide polymorphisms relative to one another [14]. Therefore, direct specific primer-pair based PCR sequencing from both strands on the purified amplified U38(POL) (480-bp), U71-gM (712-bp), and U77(HEL) (914-bp) loci was carried out on all of these new Indian necropsy samples as well. For U60(TERex3) a larger 742-bp locus was used rather than the 320-bp region previously employed [9, 10]. The results revealed that they all showed typical EEHV1A rather than EEHV1B DNA sequence patterns that in some cases were identical to other samples, but mostly displayed just a small number of novel nucleotide polymorphisms relative to one another. In contrast, the IP93 sample, which had proven to be the only Indian B-subtype found at three of these loci in our previous studies [20], also revealed a typical B-subtype pattern when newly tested here at the U77(HEL) locus. Across all four conserved loci, IE93 differed from the prototype EEHV1A (Kimba) strain by 103-bp, but differed from the prototype EEHV1B(Emelia) strain by just 12-bp, whereas EEHV1A(Kimba) and EEHV1B(Emelia) differ here by 100-bp.

To unambiguously demonstrate that individual EEHV1 samples represent distinct and epidemiologically independent strains, it is necessary to evaluate several additional PCR loci representing more variable genes [5, 20, 32]. We previously identified and used two such hypervariable gene loci, namely U51(vGPCR1) (805-bp) and U48(gH-TK) (819-bp), but currently have also now routinely added two more new hypervariable loci E5(vGPCR5) (966-bp) and E54(vOX2-1) (844-bp). The first two loci still reliably identify the well-conserved chimeric subtype EEHV1B genotypes here at both the DNA and protein levels, but also resolve all other EEHV1 strains into clusters of very closely related members of four to five other major non-B subtype patterns. Importantly, there is no linkage found to patterns across the rest of the genome between the various non-B subtypes at these latter two loci. U51(vGPCR1) contains just a single small predominant hypervariable domain found between nucleotide positions 540 to 560 that defines the principle A, B, C, D and E patterns at the amino acid level. In addition, there are many DNA polymorphisms that give additional subgrouping here, including the E versus E/A patterns in particular [20]. However, within the U48(gH-TK) locus the EEHV1B subtypes are hugely diverged from the others here by up to 35% at the aa level and the A, C, D, E and F subtypes themselves are diverged from each other by between 7 to 15% at the aa level. Once again, this expected result of a typical B-subtype pattern for IP93 applied to U51(vGPCR1) in the earlier studies, and the newly added data here for IE93 U48(gH-TK) also proved to be a very typical B-subtype pattern, with both being just one bp different from EEHV1B(Emelia).

In contrast, the new E5(vGPCR5) and E54(vOX2-1) loci mapping towards the ends of the genome outside of the conserved core segment both fail to either identify or link to the characteristic classic chimeric core EEHV1A versus EEHV1B subtype patterns. Instead, the E5(vGPCR5) locus falls into three distinct subtype groups referred to as A, B and C where the divergence is largely confined to a 200-bp central block with more than 25% polymorphic nucleotide positions (including a five aa deletion in the A strains). These three E5(vGPCR5) subtype clusters do not show linkage to the subtype patterns found at any other loci examined. Furthermore, the E54(vOX2-1) locus displays up to 15% nucleotide and aa divergence levels that distinguish amongst most but not all independent EEHV1 strains (including the otherwise largely invariant chimeric EEHV1Bs). However, the E54(vOX2-1) patterns obtained are too complex to easily categorize into recognizable subtype clusters. Nearly complete data are included for all four hypervariable loci from each of the 13 new cases and we have also added results for the E5(vGPCR5) and E54(vOX2-1) loci as well for many of the previous older India cases. Overall, complete data at all eight PCR loci tested were obtained for nine of the new cases, with just one locus missing for IP152 and IP213, whereas only three loci were successfully amplified and sequenced for case IP169 (which was derived from a heavily putrefied tissue sample).

### Multiple new independent EEHV1A strains

All good quality data from the resulting PCR DNA sequence analyses at up to eight non-adjacent PCR loci for each of the new Indian samples (totaling 6,092-bp when complete) plus selected additional loci for several of the old India samples has been assembled, edited and annotated then deposited in GenBank in a total of 110 new files plus five expanded files, whose GenBank accession numbers are listed together with sizes and map coordinates of all eight PCR loci in **Table 2**. A summary of the subtyping data results obtained for all 22 Indian cases examined is presented in **Table 3**, although only a plus or minus result is recorded for the E54(vOX201) locus. These data include newly added or revised subtype designations for the old India cases within the U71-gM, U77(HEL) and U60(TERex3) loci.

**Table 2:** Map coordinates and GenBank accession numbers of all sampled PCR loci.

Locus	Gene-Protein Names	Size DNA (bp)	Size Protein (aa)	Fig No	Genome Coords [NAP23, Kimba]	GenBank Batch DNA Acc Nos
1a	E5(vGPCR5)	962		2f	6131–7069	MF57902-40,44,46,47
1b	E5(vGPCR5)		308	3c	6161–7069	
2	U38(POL-B)	486		2a	78391–78881	*JX011032-38
						MF579054-57,63–70
3a	U48(gH-TK)	850		2f	107059–106214	*JX011039-46
						MF579091-94,98–103
3b	U48(gH-Nterm)		153	3b	105830–105379	
4a	U51(vGPCR1)	677		2e	109398–110062	*JX011047-55
						MF579091-94,98–103 MG020486
4b	U51(vGPCR1)		225	3a	109398–110062	
5a SHORT	U60(TERex3)	316		2c	123686–124042	*JX011056-62
5b LONG	U60(TERex3)	742		2c	123686–124427	MF579071-74,78,79, 82–90
6	U71-U72 (gM)	651		2b	132971–133622	*JX011063-71
						MF579104-107,113–119
7	U77 (HEL)	952		2d	140919–141870	*JX011072-79
						MF579120-123,129–135 MG020487
8a	E54(vOX2-1)	854		2h	174319–175173	MF464882-MF464899
8b	E54(vOX2-1)		295	3d	174319–175206	

*From Zachariah et al (2013). Note: Accession Nos for IP240 set are MG020478-485.

**Table 3:** Summary of PCR locus gene subtyping results.

Locus Name	E5	vGPCR1	U71-gM	POL	TER	gH-TK	HEL	vOX2-1
**New India Cases:**								
IP143 = Kozikode1	C1	E	A2’	A	A1	F	C1	*3
IP152 = Nilambur	B1	A2	A2	T960C	A1	-	C2	*5
IP164 = Muthanga	B2	E/A	A7	T960C	A3	E	C1	+
IP165 = Thirunelli2	C2’	E/A	A2	T960C	A1	E	C1	*7
IP169 = Khazagod	B1	-	-	-	A1	-	-	+
IP183 = Kozikode2	C1	D1	A2	T960C	A5	C1	D	+
IP200 = Konni1	C2	E	A1	T960C	A5	F	C3	*4
IP208 = Munnar	A	E/A	A1”	A750G	D	D	C3	*4
				G1041A				
IP212 = Konni2	C1	E	A4	A	A1	F	C1	+
IP213 = Tadoba	B1	-	A2’	A	A1	F	A1	*6
IP222 = Periyar	C1	E	A2	A	D	D	C2	+
IP239 = Tholpetty	C2’	A	A2	T960C	A	C1	C3	*7
IP240 = Periyar2	C1	E	A1	A	A5	F	C1	*1
**Old India Cases (with updates)**								
IP01 = Muthanga1 “Sunni”	-	A2	A2	T960C	(A)	A2	C3	-
IP05 = Thekkady	-	E	A4	A	(C)	F	C1	-
IP06 = Kodanad1”Nirangen”	B	E	A1”	A	D*	C3	C2	*1
IP07 = Kodanad2”Aswathi”	B1	E	A1”	A	D*	C3	C2	*1
IP11 = Pathiri1	B1	E/A	A2	T960C	A1*	C3	C3	+
IP43 = Vandalur “Chellama”	B1	D1	A2	A	A1*	C1	C3	-
IP60 = Thrissur1	-	D2/A	A3	A	-	D	C3	-
IP91 = Thirunelli1	-	A2	A2	T960C	(A)	D	C1	-
IP93 = Thrissur2	B1	B1	B	B1	B2*	B	B	*2
**Other Asian Range Country Cases:**								
SP01 = Sumatra1	-	D2	A6	A	(A)	-	C4	-
SP02 = Sumatra2	-	D2	A6	A	(A)	-	C4	-
MP01 = Myanmar1	B	-	A4	A	A	D	A1	*3
MP02 = Myanmar2	B	-	A4	A	C	D	C2	+
MP03 = Myanmar3	C	E	A5	A	A	D’	A2	*2

A’, A”, D’ etc indicates distinctive minor variants. () short version of the TER locus.

*denotes that the TER data here was expanded over that presented previously.

- = Not done. For POL, an A alone indicates the classic consensus pattern with no polymorphisms. For E54(vOX2-1), + = Novel Sequence; *1 = Identity between IP06, IP07 and IP240; *2 = Identity between IP93 and MP03; *3 = Identity between IP143 and MP01; *4 = Identity between IP200 and IP208; *5 = Identical to NAP80; *6 = Identical to NAP63 and *7 = Identity between IP165 and IP239.

**Fig 2:**
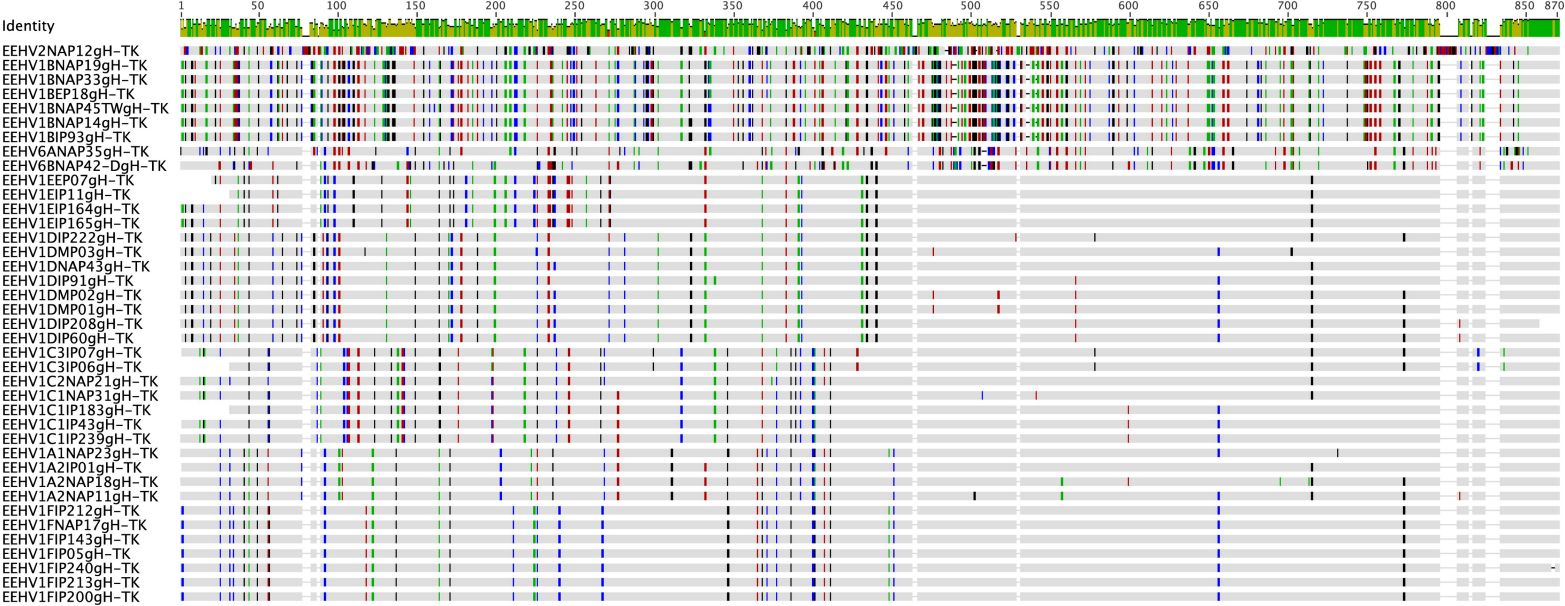
Nucleotide sequence polymorphism chart for Indian HD cases across the EEHV1 U48(gH)-TK PCR locus. The linear graphic charts were generated in Geneious 5.4.6 from alignments in MUSCLE followed by MEGA5 Bayesian phylogenetic trees dendrograms comparing the Indian *Proboscivirus* case (IP#) results with available data for three Myanmar (MP#) cases (to be reported on elsewhere) plus representative North American (NAP#) and European (EP#) cases. A total of 39 nucleotide sequences are presented including the prototype EEHV2(NAP12) genome as outgroup. Assigned subtypes are given within the designated codenames listed on the LHS of the figure panels.

DNA level difference data for the U48(gH)-TK PCR locus are presented in a graphic chart format in **Fig 2** and for the other seven PCR loci in **S1 to S7 Figs.** These diagrams show the relative positions of all nucleotide polymorphisms across each locus for our entire set of Asian case samples, together with available data for the five Myanmar and Sumatra cases that will be described elsewhere (Stremme C. et al, submitted; Oo Z.M. et al, submitted). For comparison, data for the equivalent regions of the intact prototype EEHV1A(Kimba) and EEHV1B(Emelia) genomes (GenBank Acc Nos. KC618257 and KC462164) is included for all loci. The charts also contain matching data for between seven and 12 other selected EEHV1 examples, encompassing all known subtypes, from amongst either European (EP#) or North American (NAP#) samples.

**Fig 3:**
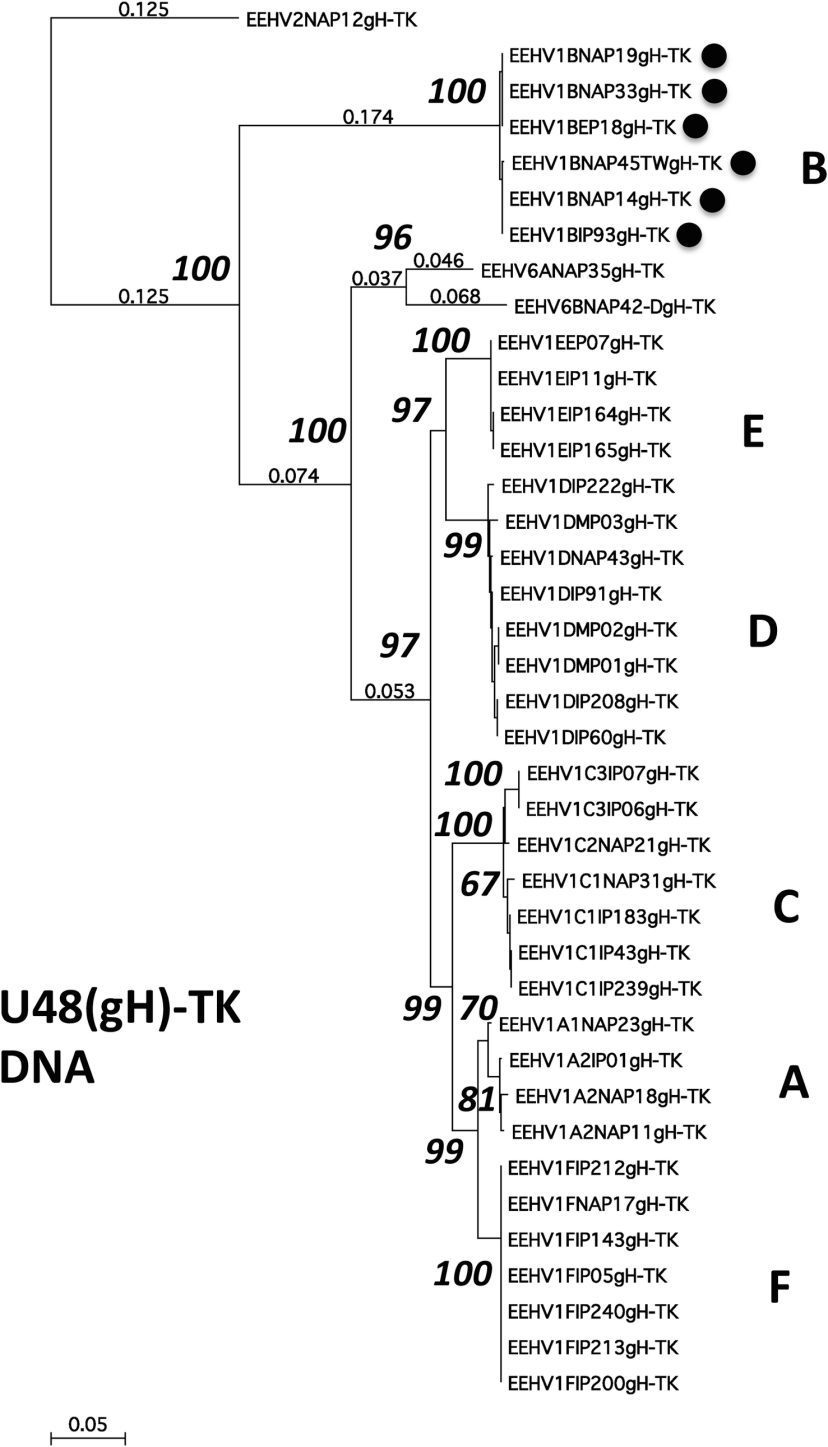
Nucleotide level phylogenetic tree for the EEHV1 U48(gH)-TK PCR locus (871-bp). Bayesian linear phylogenetic trees generated in MEGA5 from the same aligned nucleotide data sets as in Fig 2. The evolutionary history was inferred by using the maximum likelihood method based on the Tamura3-paramer model with bootstrap consensus tree. The analysis involved 39 nucleotide sequences with 758 positions in the final dataset. Data from EEHV2(NAP12) was used as the outgroup. The tree is drawn to scale with branch lengths measured as the number of substitutions per site. Where space permits some fractional branch length values are provided (smaller not bolded numbers). The percentage of replicate trees in which the associate taxa clustered together in the bootstrap test are shown next to the branches (large bolded numbers). All six examples that have classic EEHV1B core chimeric domain (CD) features (see **Fig 1**) are marked with solid circle motifs. Similar DNA trees were also generated in MEGA7 by the neighbor joining, maximum evolution and UPMGA methods but all gave essentially identical structures to the maximum likelihood result shown.

**Fig 4:**
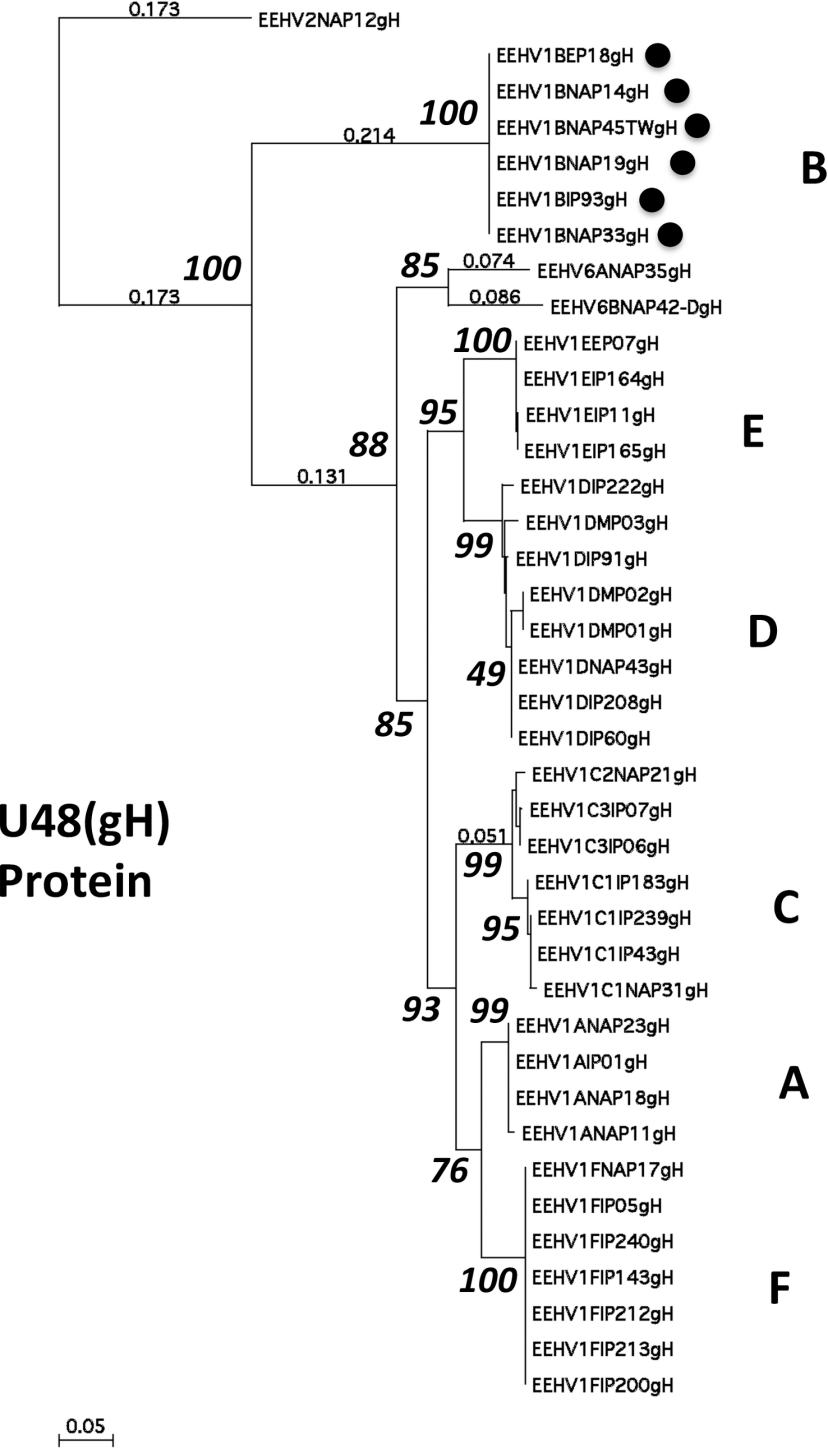
Selected protein level phylogenetic tree for the EEHV1 U48(gH) PCR locus (179-aa). Bayesian linear phylogenetic trees generated from translated amino acids in MAGA5.4.6 using similar aligned nucleotide data sets as in Figs 2 and 3, except that only the glycoprotein-H protein coding data was included from the U48(gH-TK) locus. The evolutionary history was inferred by using the maximum likelihood method based on the JTT matrix-based model with bootstrap consensus tree. The analysis involved 39 nucleotide sequences with 163 positions in the final dataset with EEHV2(NAP12) as the outgroup. The tree is drawn to scale with branch lengths measured as the number of substitutions per site. Where space permits some fractional branch length values are provided (smaller not bolded numbers). The percentage of replicate trees in which the associate taxa clustered together in the bootstrap test are shown next to the branches (large bolded numbers). All six examples that have classic EEHV1B core chimeric domain (CD) features (see **Fig 1**) are marked with solid circle motifs. Similar protein trees were also generated in MEGA7 by the neighbor joining, maximum evolution and UPMGA methods but all gave essentially identical structures to the maximum likelihood result shown.

DNA level phylogenetic trees using the same DNA sequence data for the U48(gH)-TK PCR locus are presented in **Fig 3** and for each of the other seven PCR loci in **S8 to S14 Figs**. With the single exception of the E5(vGPCR5) locus, corresponding regions from one or more of either the prototype EEHV2(NAP12, Kijana), EEHV5(EP24, Vijay) or EEHV6(NAP35) genomes were available to be used as comparative or outgroup data for both the charts and phylogenetic trees. Finally, similar protein level phylogenetic trees illustrating the results for U48(gH) are presented in **Fig 4** together with the results for the other three most hypervariable loci U51(vGPCR1), E5(vGPCR5) and E54(vOX2-1) in **S15 to S17 Figs.** Note that only data for the gH coding region (not the adjacent and overlapping TK coding segment) is included from the U48(gH)-TK PCR locus here. Nevertheless, the DNA and protein level trees for this locus are virtually identical with the distinction between the A1 and A2 subtypes within Fig 3 but not within Fig 4 being the only significant difference. For the single Indian EEHV1B(IP93) strain, **Table 4** also presents direct nucleotide number differences at each locus compared to the prototype EEHV1A(Kimba) and EEHV1B(Emelia) strains, which total 369 and 95-bp respectively.

**Table 4:** Summary of nucleotide differences for IP93 versus prototype EEHV1A(Kimba) and EEHV1B(Emelia).

	IP93 vs Emelia	IP93 vs Kimba	Kimba vs Emelia
E5	36	69	78
vGPCR1	2	36	34
U71-gM	3	47	48
POL-AB*	0	15	15
TER*	8	22	17
gH-TK	3	233	231
HEL*	1	19	20
vOX2-1	42	31	22
Total	95	369	365
Core conserved*	12	103	100

Overall, the results obtained for the new Indian samples revealed a set of 13 novel and easily distinguishable EEHV1A strains that display similar overall levels of variability to those observed across these same loci amongst all other North American, European and Asian cases that we have examined previously. Although just a few loci from several of the new strains did prove to be identical across two or more samples, none were identical across all loci. Many subtle differences from most or all previous samples evaluated were evident, but there were no new subtypes or otherwise strikingly novel patterns revealed. The pair of cases reported previously to occur three days apart at the same Indian orphan camp in calves that had been born and orphaned in two different locations within the Western Knats (IP06 Nirangen and IP07 Aswathi) remains the only pair of completely identical samples seen so far in India [20]. However, whilst the three Myanmar cases all proved to be distinct and novel strains not seen before (although two have partial identity), the two Sumatran cases SP01 and SP02 (which similarly occurred at the same camp at nearly the same time), again also proved to harbor identical strains at all loci tested. In both paired Asian cases, the identical sequence patterns were even found across the hypervariable E54(vOX2-1) locus, although that result has now also been observed amongst several pairs of otherwise distinct epidemiologically independent strains (see below). Finally, the old IP93 case remains as the only example of a classic EEHV1B subtype strain [14, 20] identified so far in either India, Myanmar or Sumatra, although several more have been recognized in the studies from Thailand [23, 33]. The IP93 sample does have the novel feature of five additional unique polymorphisms compared to all the other EEHV1Bs examined so far within the U60(TERex2) locus leading us to designate it as the first example of a B2-subtype here.

### Subtype patterns within the four most conserved PCR loci

The first four difference charts presented in **S1 to S4 Figs** show the patterns of nucleotide variation found across the standard conserved U38(POL), U60(TERex3), U71-gM, and U77(HEL) PCR loci from all Indian cases compared to representative and prototype examples from our earlier studies amongst North American and European cases, as well as five more from other Asian range countries. The variations here that were found to be common to all examples of the classic B-subtype chimeras (denoted by solid circles) at each of these four loci compared to the remaining far more numerous non-B-subtype versions all fall into the range of 3 to 5% differences at the nucleotide level. Whilst many epidemiologically unrelated strains display identical sequence patterns to one another at certain single individual loci this never continues across multiple loci. Nevertheless, there is also a sufficient level of sporadic polymorphisms even amongst members of the same subtype clusters, such that most strains can be distinguished from one another at many or all loci. The two exceptions to this rule among Asian samples as mentioned above (IP06 and IP07) plus SP01 and SP02) now bring the overall total to six known pairs of epidemiologically connected cases of HD that occurred at the same facility at close to the same time and are identical to one another at all loci tested.

Except in U38(POL), the non-B strains generally split into one or more additional consistent slightly diverged patterns that we usually designate as either A or C-subtypes (or occasionally also D) and that can sometimes be differentiated further as A1, A2, A3 etc. or as A/B or C/D if apparently recombinant. For example, at the U71-gM locus, where the A and B subtypes display 32 common polymorphisms, we also define a third C-subtype group, which consists of just two North American examples (NAP11 = Kumari and NAP17 = Singgah) that are clearly intermediates between the A and B cluster patterns with either 20 or 25-bp differences from both. In another (but rare) example, just a single consistent nucleotide difference T450G occurring in over one third of the strains was considered sufficient to define a C- versus A-subtype for the U77(HEL) locus, despite there also being several other more randomized single polymorphisms present. That same position has a T450A polymorphism in the B- and D-subtypes.

The simplest pattern occurs at the U38(POL) locus, where amongst the Indian and several selected prototype examples the predominant feature is just the subtype-A versus subtype-B diaspora, with 14 common nucleotide polymorphisms between them, plus a total of ten additional rare single point mutations. One of the latter, a T to C at position 960 occurs in nine of the India samples (**Table 3**), but has not been seen elsewhere. Similarly, for U60(TERex3), the situation is slightly more complex in that there are again 13 subtype-B defining polymorphisms common to IP93 and all but one of the classic B-subtype chimeras. Finally, the U77(HEL) locus gives a consistent pattern of 19 common polymorphisms across all but one of the classic subtype-B examples shown, including IP93, with a total of 18 additional strain-specific mutations amongst the non-B subtypes set. The single exception is NAP19 (= Haji) which instead contains an additional recombinant chimeric A-subtype segment across three adjacent loci, including U60(TERex3), U71/gM and U77(HEL), but remains a classic B-subtype on both the left and right sides of that region [14]. Several other examples of both the U77(HEL) and U60(Terex3) loci also have a small number of other unusual divergent features (including in a few cases some partial subtype-B-like character), and are designated here as subtypes -C or -D. This includes the unusual example of a D-subtype for the U77(HEL) locus in IP183, which has some partial B-like characteristics and similarly for four Indian examples of subtype-D within the U60(TERex3) locus.

### Detailed evaluation of the results at each the four most hypervariable loci

For a fuller evaluation of the other four much more variable PCR gene loci, we generated DNA level difference charts, together with both DNA level and protein level phylogenetic trees for each. However, just the most dramatic example of the U48(gH-TK) locus is presented directly in the text as **Fig 2, Fig 3** and **Fig 4,** respectively. Equivalent results at the other three hypervariable loci are shown in **S5 to S7 Figs, S12 to S14 Figs and S15 to S17 Figs**.

At the U48(gH-TK) DNA locus, the new India samples included 2x C1, 2x D, 2x E and 5x F subtypes compared to 1x A2, 1x C1, 2x C3, 2x D, 1x E, 1x F and 1x B for the old India set. At this locus as well, the three Indian Es were identical, and again as shown in Figs 2 and 3, they match the prototype E example from EP07, whereas the four Ds exhibit just three polymorphisms amongst them. Similarly, the six Fs proved to be identical to one another and to the prototype USA example (NAP17), and the same applies for the two C1s and the two C3s. Remarkably, although the India IE93 B-subtype displayed 233 out of 832-bp differences from the EEHV1A(Kimba) prototype (subtype = A) here, it proved to be almost identical to each of the other five known B-subtypes at this locus.

Interestingly, the U48(gH)-TK locus displays a dramatic internal dichotomy with the level of nucleotide divergence between all six subtypes across the first half (i.e. at positions 1 to 455 within the bulk of the U48(gH) gene segment that does not overlap with the TK gene) being much higher than across the second half encompassing the U48.5(TK) gene segment. In fact, the overall level of nucleotide polymorphism (omitting deletions) across the U48(gH) gene portion on the LHS of this locus amongst all the EEHV1 strains shown in the chart is as high as 37%, but drops to 26.6% when the B-subtype examples are omitted. In comparison, the value is 24.3% across the TK gene coding block on the RHS, but falls dramatically to only 4.5% when the B-subtypes are omitted. Across this whole locus, the six known B-subtypes are almost completely identical amongst themselves, but differ hugely from all the other EEHV1s by around 35% overall at the nucleotide level (**Figs 2 and 3**). Even the only two known examples of EEHV6 (both included in the charts) show a similar level of nearly 30% divergence from each other and all EEHV1s across the LHS U48(gH) segment, whereas they are nevertheless largely invariant across the RHS U48.5(TK) segment, whilst still maintaining even higher levels of divergence from both the EEHV1Bs and also from the entire remaining EEHV1 A to F subtypes group. Note that the protein phylogenetic tree derived from this locus in **Fig 4** encompasses only the highly variable U48(gH) coding segment not the less variable U48.5 TK segment.

For the U51(vGPCR1) DNA locus (**S5 and S12 Figs**), the new India cases included the following subtypes, 2x A2, 1x D1, 5x E and 3x E/A, whereas the old India set included 2x A2, 1x D1, 1x D2/A, 3x E, 1x E/A and 1x B. Neither group contained any A1 or C subtypes and only the original set included a single B subtype (IP93). All seven E subtype examples were identical to one another at the nucleotide level as also were the four E/As and the two D1s, but there were three polymorphic positions amongst the four A2s. Most of them also showed identity to one or more from a subset of the North American cases as well. At the protein level, even the E plus E/A subtypes proved to be identical with no polymorphisms at all across all 11 Indian examples (**S15 Fig**). No data was available at this locus for case IP213. Amongst the Indian samples shown on the chart (including the single B-subtype IP93) there are a total of 61 out of 670 polymorphic nucleotide positions overall, (excluding deletions), representing 9.1% divergence, but at this locus the major subtype-defining variations are largely confined to the five clustered patterns of aa insertions and deletions within the “hot-spot” between nucleotide positions 512 and 570. Once again, all six classic B-subtypes (including NAP19 and IP93) are both very distinctive and identical here across nucleotide positions 450 to 600, although NAP33 and NAP45TW do differ significantly from the others within the first 300-bp of this locus.

For the E5(vGPCR5) locus (**S6, S13** and **S16 Figs**), where the previously analyzed North American and European cases fall into just three roughly equally abundant subtypes (A, B or C), the new India cases yielded 1x A, 4x B and 6x C with three of the latter (IP165, IP239 and IP240) being identical C2’variants, whereas all five old Indian cases analyzed (including IP93) were B-subtypes at this locus. No data is available here for the other four old India cases IP01, IP05, IP60 or IP91. Once again, this locus shows a curious dichotomy whereby most of the subtype-specific nucleotide and nearly all of the protein variability occurs just within the RHS C-terminal segment of the protein from position 485 to 710. Despite significant (but not subtype-defining) nucleotide variation (**S6 and S13 Figs**) within the LHS segment (9.5% variation overall across the N-terminal half of the protein at positions 1 to 485) there is very little aa variation here (**S16 Fig**). However, the overall nucleotide polymorphism level reaches 21% between positions 485 to 965 and rises further up to 31% just between positions 485 and 710. The overall nucleotide divergence common to most examples of the A, B and C subtypes from the consensus here measures out at just about 4%, 2% and 3% respectively across the whole protein and is again heavily concentrated just between positions 485 to 710 (**S6 and S13 Figs**). Only a small fraction of strains within a single subtype display identity here, but they do still each closely resemble one of the appropriate selected prototypes given from amongst the North American cases. Whilst four of the six classic B-prototypes shown (NAP14, NAP33, NAP45TW and the Indian IP93) do happen to fall into the E5(vGPCR5) B-subgroup here, there are six other classical non-B chimera examples from India that also fit into the same B-subtype group here, clearly showing that there is either no linkage or at most just a highly scrambled residual level of linkage to the classical EEHV1A versus EEHV1B chimerism patterns in E5(vGPCR5).

Finally, for the E54(vOX2-1) locus, data was obtained for all 13 new India cases and four of the old India cases as shown in **S7** and **S14 Figs** at the DNA level and in **S17 Fig** at the protein level. There is no data here for IP01, IP05, IP60 or IP91 and although we do have about 300-bp for one strain of EEHV6 (NAP42-D) (shown in **S7** Fig only) that data covers a segment that is too small to allow meaningful phylogenetic comparisons in the matching DNA and protein trees (**S14** and **S17 Figs**). Therefore, data for EEHV5 (Vijay) is used instead as the outgroup for E54(vOX2-1) in **S14** and **S17 Figs**. Remarkably, although the 290-aa double immunoglobulin-like domains of this viral version of this captured host cell protein remain highly conserved at the aa level (with between 90 to 97% identity to the equivalent *Elephas maximus* encoded OX2 or CD200 host protein), it is still highly variable at the DNA level, exhibiting up to 35% overall polymorphic nucleotide positions occurring amongst the 29 independent EEHV1 case samples illustrated in the DNA polymorphisms chart in **S7 Fig** and DNA tree in **S14 Fig**.

Despite showing clear evidence that the E54(vOX2-1) locus does display extensive but complex subtyping effects, with short patches of matching nucleotide similarity occurring between some strains, the resulting patterns are far too scrambled (presumably by homologous recombination) to be able to cleanly define any specific subtype clustering. Nevertheless, there are multiple examples where segments of several different strains display obvious high levels of partial resemblance or identity, as well as several segments where the divergence reaches similar high levels to that between EEHV1A and EEHV2. The most notable of these “hot-spots” are from nucleotide positions 10 to 160 in NAP45TW, 600 to 680 in NAP14, 500 to 600 in IP143, 500 to 650 in IP164, IP165 and IP213 plus 590 to 660 in IP212. Interestingly, one of the Indian strains IP11 shows nearly the same level of divergence from all other EEHV1 strains as for EEHV5 and EEHV6 (**S7 Fig**) across almost the whole length of the vOX2-1 gene, but switches to being identical to both IP212 and NAP45TW over the final 200-bp. As an example of one of the highest levels of diversity seen across the E54(vOX2-1) locus amongst EEHV1 strains, IP06 and IP11 differ here by 136 out of 850-bp (15%) at the nucleotide level.

When just IP11, IP212 and NAP45TW are omitted from the analysis, the total level of polymorphisms across the E54(vOX2-1) genes in EEHV1 strains drops from 285 out of 806 bp (35%) to 199 out of 806 bp (25%). Furthermore, the high preference for displaying synonymous rather than non-synonymous aa changes here is so extreme that across one 114-bp section from positions 489 to 603 there are a total of 31 out of 34 successive codons (excluding gaps) that have polymorphism at the wobble position with only seven other non-wobble positions having polymorphisms within the same region. Unexpectedly, like E5(vGPCR5) above, this locus from close to one end of the novel segment of the genome does not show any obvious linkage to the conserved core region EEHV1A versus EEHV1B chimerism patterns. In fact, the vOX2-1 sequences from all six representative examples of classic core B-subtype genomes NAP14, NAP19, EP18, NAP33, NAP45TW, NAP49 and IP93 (as denoted by solid circles) that are included in the DNA difference chart (**S7 and S14 Figs**) and protein tree (**S17 Fig**), proved for the most part to be highly diverged from one another, whilst also showing no significantly greater than usual differences from any of the non-B subtype examples.

### Comparison with other strains examined in detail worldwide

Zachariah et al [20] pointed out that the seven distinctive EEHV1A strains examined initially encompassed a similar wide range of genetic variants as encountered previously in both lethal and surviving hemorrhagic disease cases studied from European and North American zoos. We did note then that three Indian strains had a common unique T960C polymorphism in the POL locus that had not been seen elsewhere, but otherwise the numbers were too small to make any interpretations about whether there were any overall localized trends observable amongst the Indian cases. However, now that we have moderately extensive data from a total of 27 independent Asian strains, including five from Myanmar and Sumatra (as summarized in **Table 2**), several additional common trends have indeed emerged. Firstly, amongst the four conserved region loci, the unique non-syngeneic T to C polymorphism in U38(POL) has now been found in nine of the 20 South India EEHV1 examples studied, but so far in just one other (NAP31) from a total of 45 other EEHV1 examples that we have evaluated from outside of India, and not in Thailand either. Somewhat similarly, although the difference between the assigned A and C subtypes in U77(HEL) are based solely on a single T450G polymorphism in the latter, 17 of the 20 cases from India fall into the C group (plus one each of A, B and D) with 3x C and 2x A from other parts of Asia, whereas amongst the combined European and North American sets there were instead 13x A, 5x B, 15x C and 2x D and in Thailand there were 3x A, 2x B, 7x C and 3x D.

**Fig 5:**
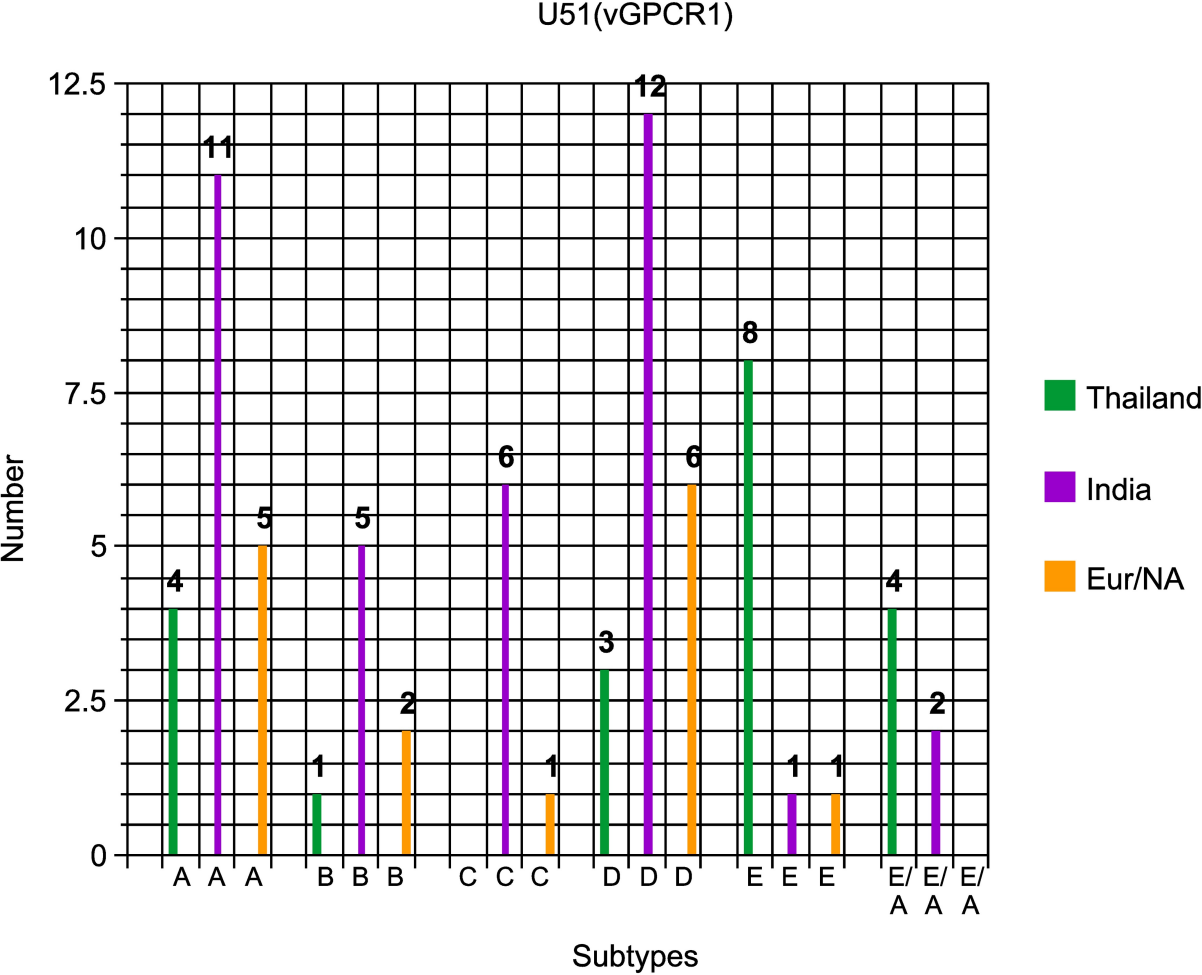
Distinctive subtype distribution patterns for the EEHV1 U51(vGPCR1) PCR locus. The numbers of positive EEHV1 examples of each subtype found amongst both the India and Thailand cases compared with those from Europe and North America combined are presented in a bar graph format as indicated.

Secondly, no fewer than 12 out of the 20 different South Indian EEHV1A strains evaluated at the U51(vGPCR1) locus (60%) have proven to be either E or E/A-subtypes, whereas only four out of 34 (12%) from European and North American EEHV1A examples were of these subtypes (plus one from Myanmar). In contrast, 11 out of the 13 EEHV1A cases from Thailand examined at this locus [23] were instead A or D-subtype variants in U51(vGPCR1) with just a single example each of C and E. In comparison, just three from South India and both Sumatran cases evaluated at this locus have been D variants compared to 13 of the 34 European plus North American examples (38%). Similarly, whilst there have been four A subtype vGPCR1 examples detected amongst the Indian cases, there were 11 A-subtype vGPCR1s found in Europe or North America. On the other hand, no examples of a C-subtype vGPCR1 have been found either within India or anywhere else in Asia in our studies, and with just the single example from Thailand, whereas there have been six C-subtypes found so far amongst European and North American cases. These large differences in the distribution of U51(vGPCR1) subtypes between India and Thailand as well as in comparison to North American plus European examples are illustrated graphically in **Fig 5**.

**Fig 6:**
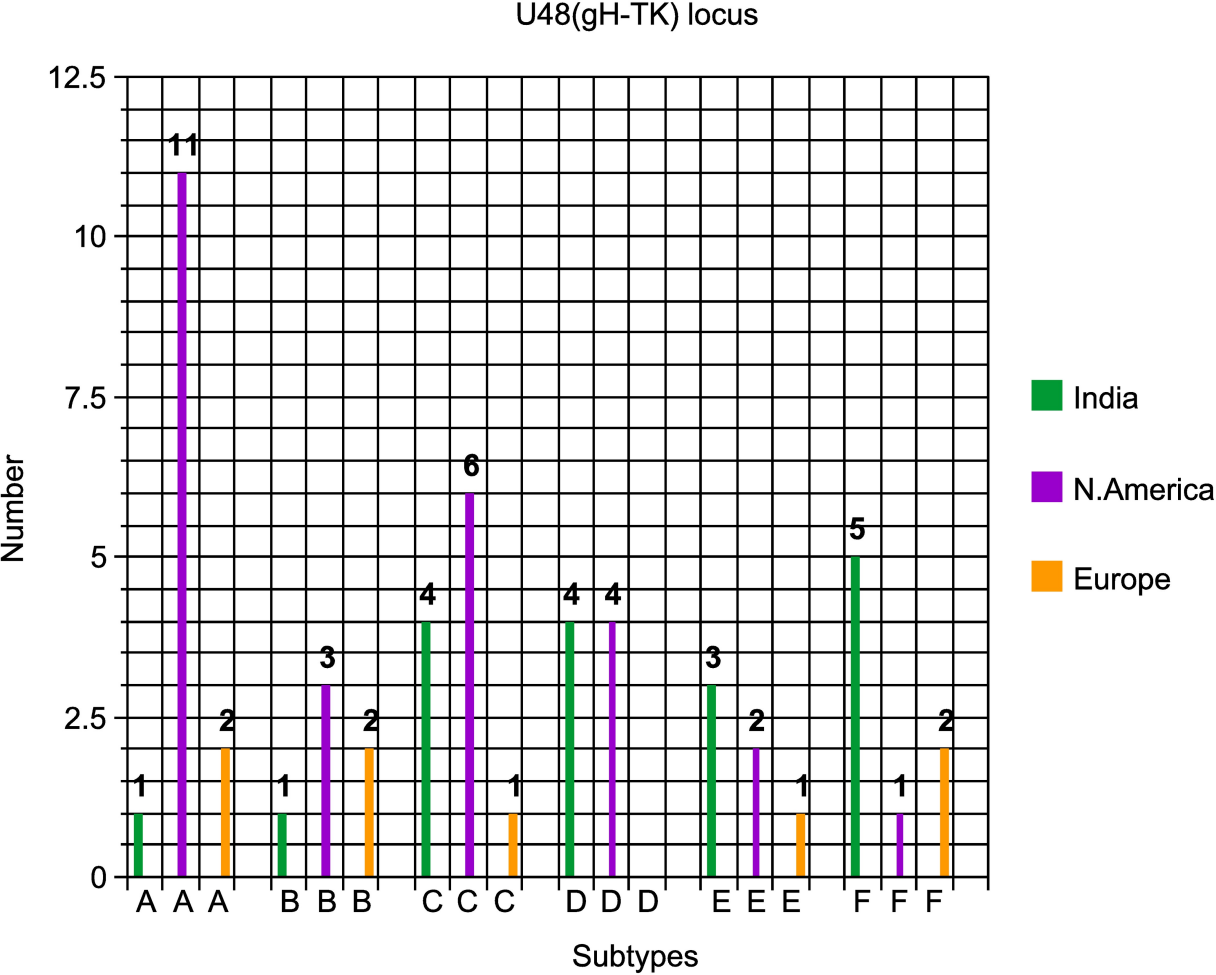
Distinctive subtype distribution patterns for the EEHV1 U48(gH)-TK PCR locus. The numbers of positive EEHV1 examples of each subtype found amongst the India cases compared with those from Europe and North America are presented in a bar graph format as indicated.

Thirdly, amongst the Indian EEHV1A cases, four examples of the U48(gH) locus were C-subtypes, plus 4x D, 3x E, 5x F but just 1x A and 1x B, with all three from Myanmar being D-subtypes. This compares to 14x A, 6x C, 4x D, 3x E, 2x F and 5x B-subtypes here from a total of 34 European and North American cases, but there is no data available for the U48(gH) locus nor for E5(vGPCR5) or E54(vOX2-1) from Thailand. Again, the difference in subtype distribution patterns between the India cases and the examples evaluated by our group from both North American and European examples is illustrated graphically in **Fig 6**.

**Fig 7:**
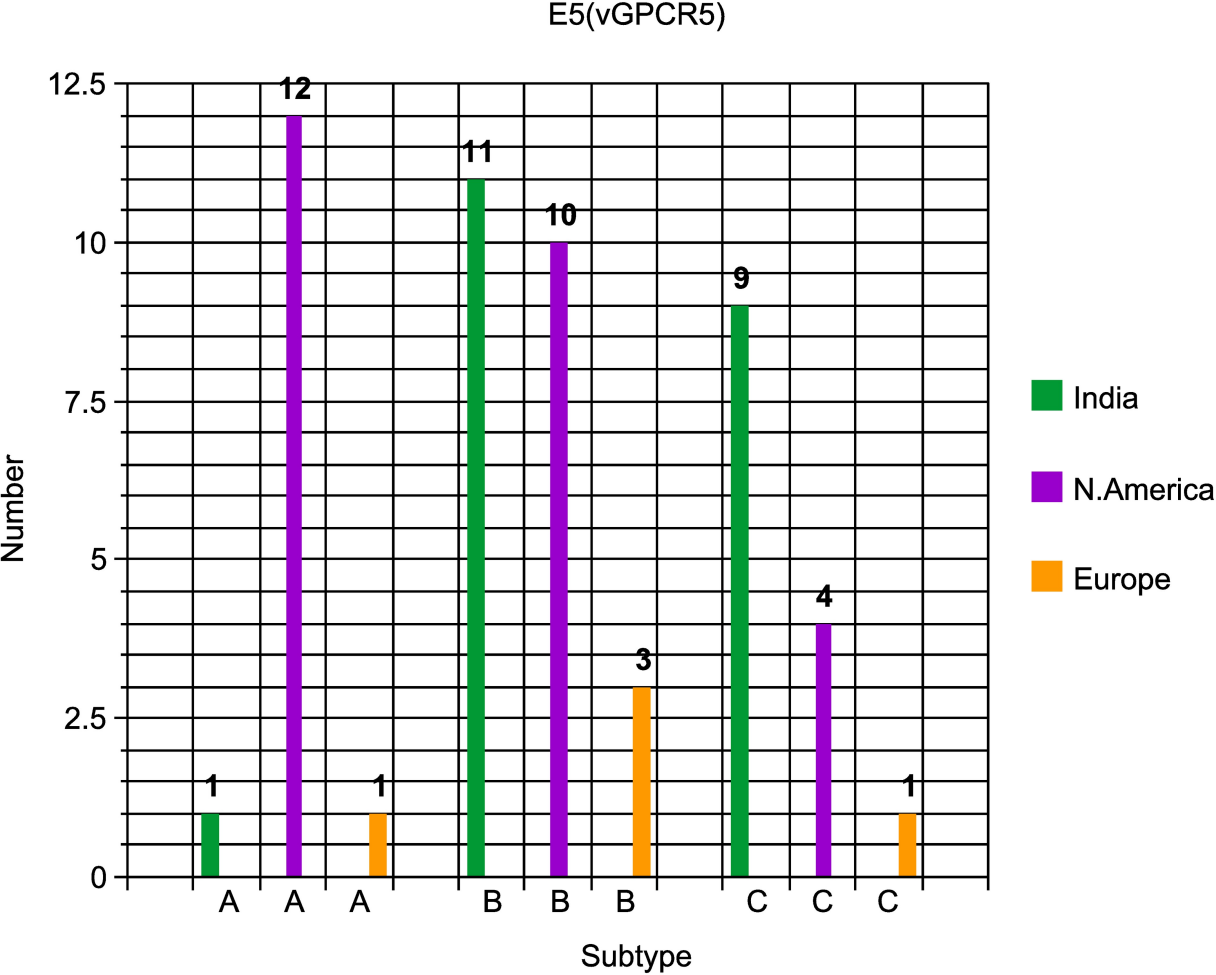
Distinctive subtype distribution patterns for the EEHV1 E5(vGPCR5) PCR locus. The numbers of positive EEHV1 examples of each subtype found amongst the India cases compared with those from Europe and North America are presented in a bar graph format as indicated.

Fourthly, for the E5(vGPCR5) locus, our studies with European and North American cases revealed that they split up into three distinct subtype clusters, with a total of 12 being defined arbitrarily as A-subtypes (including the Kimba and Raman prototypes), 14 being B-subtypes and six being C-subtypes, including the classic chimeric EEHV1B prototype strain EP18 = Emelia). As shown in **Fig 7**, in contrast to the E5(vGPCR5) subtype distribution patterns found in North America and Europe, the 21 India and Myanmar cases evaluated here included instead 11 B-subtypes and nine C-subtypes, with just one A-subtype being detected (IP208). Again, several examples of non-epidemiologically related localized identity occurred amongst the India cases, with the most notable being the three identical cases of C2’ strains here. Amongst the six non-Asian chimeric EEHV1B strains, four were B-subtypes at this locus, with one each being A and C subtypes and the single Indian IP93 EEHV1B strain was also a B-subtype here. However, nine other examples of B-subtypes in E5(vGPCR5) were from strains that have classic EEHV1A chimeric patterns.

### Multiple examples of identical DNA sequences in E54(vOX2-1) and the other hypervariable loci from unrelated independent HD cases

Finally, returning to the U54(vOX2-1) locus, it was quite remarkable to find that several otherwise unrelated Indian genomes exactly matched to one of the Asian or even North American genomes at this locus only. As expected, the six known epidemiologically related identical pairs occurring at nearly the same time at the same facility, including IP06 plus IP07 from India, SP01 plus SP02 from Sumatra, EP20 plus EP21 from the United Kingdom and EP24 plus EP25 from Germany, as well as both of the surviving NAP33 plus NAP34 and NAP39 plus NAP40 case pairs in the USA were indeed identical at all loci tested. But surprisingly, five pairs of non-epidemiologically related Asian samples, namely IP06/IP07 plus IP240, IP165 plus IP239, IP200 plus IP208 and IP143 plus MP01, as well as IP93 plus MP03 proved to be identical here also. In addition, two different Indian samples IP152 and IP213 formed identical pairs here with recent even more obviously unconnected USA samples NAP80 and NAP63 respectively. However, for all five of these latter groups of matching pairs within the U54(vOX2-1) locus, the identity occurred only at this one locus and their genomes differed extensively elsewhere. Similar situations of localized identity amongst E54(vOX2-1) loci only have also been observed amongst seven of the 34 apparently epidemiologically unrelated cases examined previously from North America namely the NAP30 plus NAP73 pair, as well as the NAP17 plus NAP80 pair, and also for three other identical examples found in cases NAP29, NAP45TW plus NAP75.

Whilst the four conserved PCR loci do also all exhibit many examples of localized identity at single PCR loci among unrelated sample pairs, the phenomenon was more unexpected and seemingly more dramatic within the hypervariable loci and especially so for E54(vOX2-1). However, the other three hypervariable loci do also show many examples of the same effect (even just amongst Asian samples). For example, in U51(vGPCR1), all eight Asian subtype-Es were identical to one another at the nucleotide level (and just one bp different from the USA prototype NAP13). Similarly, three unrelated Indian subtype-E/As (IP164, IP165 plus IP208) were identical, as also were two Indian subtype-Ds (IP43 plus IP183) and two Indian subtype A2s (IP91 plus IP239). For the E5(vGPCR5) locus, the same applies to IP165, IP239 plus IP240 (subtype-C) as well as four other pairs including IP143 plus IP222 (subtype-C), IP93 plus IP152 (subtype-B), IP143 plus IP222 (subtype-C), IP169 plus IP213 (subtype-B) and MP01 plus MP02 (subtype-B). Finally, at the U48(gH-TK) locus all six Asian subtype-Fs (IP05, IP143, IP200, IP212, IP213 plus IP240) were identical to one another (as well as to the North American prototype NAP17), and IP43, IP183 plus IP239 (subtype-C), as well as IP11, IP164 plus IP165 (subtype-E), IP60 plus IP208 (subtype-D) and again MP01 plus MP02 (subtype-D) also all exhibited identity here.

## Discussion

Although the intact DNA genomes of two EEHV1A prototype strains and one EEHV1B prototype strain (each totaling close to 180-kb in size) have been determined and annotated [25, 26, 30], pathological samples of this type are only rarely sufficiently abundant or intact when obtained directly from necropsy tissue to apply whole genome next-generation random sequencing approaches. Nevertheless, based on the complete genome data for EEHV1A (Kimba), EEHV1A(Raman) and EEHV1B (Emelia), there are also at least 30-kb each now available from PCR sequencing of key selected loci for 13 other well-studied cases, including eight EEHV1A and five EEHV1B strains from Europe and North America [14]. Furthermore, the eight selectively sequenced PCR loci employed here provide a highly representative window into the levels of variability spanning across the whole viral genome. Overall, they encompass four well-conserved central core gene loci, as well as four hypervariable segments, one from the chimeric CD-II domain U48(gH-TK), a second from within the central core domain U51(vGPCR1) and finally two novel captured cellular gene loci from opposite ends of the viral genome E5(vGPCR5) and E54(vOX2-1). Curiously, the latter two recently added loci (unlike all six core segment PCR loci examined here) fail to connect or link in any obvious way with the major chimeric CD-I, CD-II and CD-III subtype-A versus subtype-B diaspora patterns described previously amongst all EEHV1 strains [14]. For comparative purposes, the six classic chimeric EEHV1B strains and the single known Indian EEHV1B strain (IP93) display just as great a level of variability at these two outlier loci as do the entire range of EEHV1A variants. In contrast, all EEHV1B subtype sequences mapping within the other six more centrally located PCR loci from genome segments C1 and C2 have been found to be extremely highly conserved compared to the often highly diverged and unlinked subtype patterns for their EEHV1A counterparts [30].

Overall, as described in detail in the results section, the data obtained here for the 22 Indian cases display essentially the same wide range of subtype variability as found across all cases examined previously from Europe and North America. However, for the first time some degree of differentiation in the relative frequency of subtype patterns observed between both the Indian set and the Thailand set of strains and between both Asian range country sets versus European and North American subtype patterns can be discerned. These preferential subtype pattern trends apply only at the individual PCR loci level and do not show any evidence of consistent genome-wide effects or even partial linkage across adjacent loci. That the overall subtype patterns found in Asia and Europe-North America are similar is hardly surprising considering that the extant *Elephas maximus* host populations in Western zoos obviously had recent origins as imports from multiple different Asian countries. Clearly, the introduced Asian virus strains endemic to individual source countries and herds would be expected to have both prior localized ancestral divergence associated with evolutionary drift amongst themselves, coupled with some degree of additional opportunities for genetic mixing and subsequent recombination exchanges between viruses from different Asian elephant host populations as well. But evidently the levels of subtype divergence and the patterns of localized preferential subtype enrichment are both very large and unexpectedly complex.

Unlike HIV and many RNA viruses, herpesvirus genomes generally have relatively low rates of genetic drift that have been estimated at 3 x 10^−8^ changes per nucleotide per year [34]. For genomes of 2 x 10^5^ bp in size encoding around 100 proteins this is equivalent to about one nucleotide (and amino acid) change per average-sized gene every 100,000 years and in the vicinity of 10% overall nucleotide divergence every 10 million years. These values mostly apply only to well-conserved common core genes, with many of the hypervariable genes, which often fall into multiple distinct clustered subtype lineages, apparently representing very anciently diverged versions that in some cases may even have arisen originally from rare exchange events with other closely related virus species. Evidently, large segments of both the novel and conserved segments of the different subtypes later become unlinked and scrambled after presumably rather extensive genetic exchanges between strains by homologous recombination. Even although the extant modern EEHV1 virus population as a whole clearly represents a single recombining species, the last common ancestor ages (LCA) for different segments and hypervariable genes of different individual strains consists of a complex mixture of pieces derived from multiple diverged genome types and with wide ranges of both modern and anciently acquired variations. Some of the gene subtypes observed here may have diverged by standard genetic drift mechanisms beginning tens of millions of years ago then been reassembled into complex chimeric forms by relatively rare interspecies level recombination events. Therefore, the overall EEHV1 population may be best described as consisting of a swarm or quasi-species, although (unlike HIV for example) this extremely high level of variation would not be expected to change at all within the lifetime of a single individual host animal.

Current biogeographic barriers are believed to have resulted in four major distinctive populations of Asian elephants in India located in Northern, Northeastern, Central and Southern areas, with the Southern India group being further divided into two sub-populations occupying either side of the Palaghat gap. Data from both mitochondrial DNA genome analysis and nuclear satellite marker sampling suggest that the elephant populations on either side of the Palaghat gap have been effectively separated for 120,000 years [35, 36]. All but one of the lethal cases of EEHV associated HD evaluated here within young wild or orphaned elephants came from Southern India with IP11, IP43, IP91, IP152, IP164, IP165, IP169, IP183 and IP239 representing the more northerly Nilgiri Biosphere Reserve (NBR) population and with IP05, IP06, IP07, IP60, IP143, IP208, and IP240 representing the more southerly areas of Anamalai and Periyar (A/P) in Kerala. In addition, two orphans IP200 and IP212 originated in the NBR area but were raised and died in the A/P area.

Because most HD cases are believed to be primary infections, we expect that where they died is more relevant here than where they were born. Possible correlations include seven out of eight of the POL T960C polymorphisms being from the NBR area, as well as eight out of eight of the U51(vGPCR1) subtype Es being from the A/P area, whereas three out of the four U51(vGPCR1) E/As subtypes came from the NBR area. Similarly, five out of six of the U48(gH-TK) examples came from the A/P area (plus one from Central India), whereas three out of three U48(gH-TK) subtype Es came from the NBR area. However, most other subtype-specific features appear to have scrambled or mixed up locations. Therefore, overall, we have not been able to discern any clear patterns of preferential localization of more closely related EEHV1 strains to either side of the Palaghat gap, indicating that (perhaps with just a few exceptions) these numerous anciently-derived chimeric patterns were most likely largely randomized prior to formation of that new biogeographical barrier and that the 120,000 years separation probably represents too short a time period for distinctive differences or linkage patterns at most locations within the viral genomes to have emerged or to become recognizable.

EEHV-associated HD remains a serious problem in Asian elephant populations worldwide [5]. With regard to the overall occurrence in Asian range countries, cases are now known from India, Thailand, Myanmar, Laos, Indonesia-Sumatra, Singapore, Borneo and Cambodia, with Sri Lanka and perhaps Vietnam being amongst the few remaining exceptions at present. A very important feature of our India results is that unlike any of the cases reported previously from Thailand (as well as those from Sumatra and Myanmar) 12 of the 22 cases evaluated occurred in wild-born calves discovered within a variety of free-ranging herds distributed across Southern India, which is the largest contiguous population of wild elephants remaining in Asia. Although it would be hard to argue that EEHV HD is of emerging or recent origin within Asian range countries, the presence and frequency of this disease raise serious concerns about plausible negative effects on the long-term survival of the highly endangered populations of Asian elephants in the wild, as well as being a major impediment to sustainable future breeding success amongst captive-born Asian elephants and rescued camp orphans worldwide.

## Materials and methods

### DNA extraction, PCR amplification and sanger DNA cycle sequence analysis

These procedures were carried out as described previously [10, 14, 20].

### DNA sequence editing, phylogenetic trees and graphics

These procedures followed those described in Zachariah et al [20], except that initial alignments were produced in MUSCLE rather than Clustal W as implemented in MacVector 12.

### PCR amplification and DNA sequencing primers used

U38(POL) Locus: Core segment, shows low level classic chimeric A versus B subtypes.

R1 LGH6711 5’-GTA TTT GAT TTY GCN AGY YTG TAY CC-3’
R2 LGH6712 5’-TGY AAY GCC GTN TAY GGA TTY ACGG-3’
L1 LGH6710 5’-ACA AAC ACG CTG TCR GTR TCY CCR TA-3’
Round 1 R1/L1 = 530-bp, round 2 R2/L1 250-bp.

U60(TERex3) Locus: Core segment, shows low level classic chimeric A versus B subtypes.

R1 LGH6671 5’-GTT TGT AGT AAA TGC CGG ATC-3’
R2 LGH2429 5’-GTC GGC TAA ATG TTC TTG -3’
L2 LGH2430 5’-GTA CGT CCT TTC TAG GCT CAC-3’
L1 LGH6672 5’-CAT GTT GTG CAG GCA CTC TTC-3’

Extended version round 1 R1/L2 = 780-bp; Short version R2/L2 = 340-bp,

U71-gM Locus: Core segment, shows low level classic chimeric A versus B subtypes.

R1 LGH6749 5’-CTA TGG GAT CCG AAC TTT C-3’
R2 LGH6750 5’-CTT TCT AAG GGG GTT TGT TGC-3’
L2 LGH6752 5’-CTA CAT GCC CAT GCA GAT AGG-3’
L1 LGH6751 5’-GAA GTC CTG CTA GCC CCY TAC-3’

Round 1 R1/L1 = 750-bp, second round 2A R2/L1 = 730-bp, second round 2B R1/L2 = 730- bp, third round 3AB R2/L2 = 710-bp.

U77(HEL) Locus: Core segment, shows low level classic chimeric A versus B-subtypes.

R1 LGH6649 5’-CCA GTC AAC GTA TAG CTC GTA G-3’
R2 LGH6743 5’-GCA AGG TRG AAC GTA TCG TCG-3’
L2 LGH7885 5’-CTG CGT GTA ACA TGT TGG C-3’
L1 LGH3198 5’-CAC AG(A/C) GCG TTG TAG AAC C-3’ (= LGH6742)

Round 1 R1/L1 = 980-bp, round 2A R1/L2 = 950-bp, round 2B R2/L2 = 680-bp.

U51(vGPCR1) Locus: Six major subtypes including distinctive classic chimeric B-subtypes.

R1 LGH7506 5’-GAT TGT GAA CGC TGT ATG CT-3’
R2 LGH7470B 5’-(GAC) AGG TGG TAC TGT ATG ATG TGC-3”
L2 LGH5200A 5’-CGT GAT ACG CTT CAA AAC ATA CA-3’
L1 LGH4963B 5’-GAC TTT CTT CGT AGC CCT CGT CTT-3’

Round 1 = R1/L1 = 910-bp; round 2A R1/L2 = 750-bp; round 2B R2/L1 = 730-bp, third round 3AB = 570-bp.

U48(gH)-TK Locus: Six major subtypes including distinctive classic chimeric B-subtypes. Maps within CD-II core chimeric domain

R1 LGH7981 5’-CT(A/G) CAT T(T/G)(A/C) CCA AAG TAT GGA AGT A-3’
R2 LGH7982 5’-CRT YTA TAT CAT CAA ARA CYT CAC A-3’
L2 LGH7984 5’-CAG CCT TCA AGC GGC ATA CAC TG-3’
L1 LGH7985 5’-GGT AGG TTC ACC TAC ATG GAA CTT C-3’

Round 1 R1/R2 = 1080-bp. Round 2A R1/L2 = 920-bp, 2B R2/L1 = 1040-bp, third round 3AB R2/L2 = 860-bp.

E5(vGPCR5) Locus: Three subtypes only, unrelated to classic chimeric A plus B subtypes.

R1 LGH8507 5'-CCG TTT ACT TGC GTA GTA GGT ACG-3' (1)
R2 LGH8552 5'-CCG ATC TAT GTA CTT ACG YTA GCG GTG-3’ (70)
L2 LGH8558 5'-CAC GTC CGT ATG TGA CAG GTA CAA TC-3' (920)
L1 LGH8508B 5'- GAC GTG TTT CCT AGG ATT GTC AAA G-3' (1000)

First round R1/L1 = 1000-bp, round 2A R1/L2 = 980-bp, round 2B R2/L1 = 930-bp.

U54(vOX2-1) Locus: Patterns are too complex to assign subtype clusters. No correlation with classic chimeric A plus B subtypes.

R1 LGH8471 5’-ATG CTT CAG AGA AAG TA AGG TAC-3’
R2 LGH9132 5’-GGT CGT AAC GCA AGA TGA GCG AG-3’
L2 LGH8506 5’-GAT GCC TTC TAC GCC ACT TGT AAC AG-3’
L1 LGH8472 5’-GTG TTG CCG CCA CGA TGC TTC TAC G-3’

Round 1A R1/L1 = 910-bp; round 1B R1/L2 = 890-bp; round 2A R2/L1 = 740-bp; round 2B R2/L2 = 720-bp

## Supporting information

S1 Fig Nucleotide sequence polymorphism chart for indian HD cases across the EEHV1 U38(POL) PCR locus (494-bp). As for **Fig 2** except for also including data for the two Sumatran cases (SP#) and the use of both the prototype EEHV2(NAP12) and EEHV6(NAP35) genome data for comparison.
(TIF)

S2 Fig Nucleotide sequence polymorphism chart for Indian HD cases across the EEHV1 U60(TERex3) PCR locus (741-bp). As for **Fig 2** except for also including data for the two Sumatran cases (SP#) and the use of both the prototype EEHV2(NAP12) and EEHV6(NAP35) genome data for comparison.
(TIF)

S3 Fig Nucleotide sequence polymorphism chart for Indian HD cases across the EEHV1 U71-gM PCR locus (646-bp). As for **Fig 2** except for also including data for the two Sumatran cases (SP#) and the use of both the prototype EEHV2(NAP12) and EEHV6(NAP35) genome data for comparison.
(TIF)

S4 Fig Nucleotide sequence polymorphism chart for Indian HD cases across the EEHV1 U77(HEL) PCR locus (952-bp). As for **Fig 2** except for also including data for the two Sumatran cases (SP#) and the use of the EEHV6(NAP35) genome data rather than EEHV2(NAP12) for comparison.
(TIF)

S5 Fig Nucleotide sequence polymorphism chart for Indian HD cases across the EEHV1 U51(vGPCR1) PCR locus (668-bp). As for **Fig 2** except for also including data for the two Sumatran cases (SP#) and the use of the prototype EEHV6(NAP35) genome data rather than EEHV2(NAP2) for comparison.
(TIF)

S6 Fig Nucleotide sequence polymorphism chart for Indian HD cases across the EEHV1 E5(vGPCR5) PCR locus (909-bp). As for **Fig 2** except that no equivalent data is available for comparison from either EEHV2 or EEHV6.
(TIF)

S7 Fig Nucleotide sequence polymorphism chart for Indian HD cases across the EEHV1 E54(vOX2-1) PCR locus (842-bp). As for **Fig 2** except for also including partial data for EEHV6(NAP42D) and intact matching data for the EEHV5A(EP24 = Vijay) genome for comparison.
(TIF)

S8 Fig Nucleotide level phylogenetic tree for the EEHV1 U38(POL) locus (949-bp). Bayesian linear phylogenetic tree generated in MEGA5 by the maximum likelihood method from the same aligned nucleotide data set as in S1 Fig and with EEHV2(NAP12) used as the outgroup. The branch distance scale and some representative distance values are given. All six examples that have classic EEHV1B core chimeric domain (CD) features (see **Fig 1**) are marked with solid circle motifs.
(TIF)

S9 Fig Nucleotide level phylogenetic tree for the EEHV1 U60(TERex3) locus (741-bp). Bayesian linear phylogenetic tree generated in MEGA5 by the maximum likelihood method from the same aligned nucleotide data set as in S2 Fig and with EEHV2(NAP12) used as the outgroup, except for the omission of five samples with data less than 80% of the length of the intact locus. The branch distance scale and some representative distance values are given. All six examples that have classic EEHV1B core chimeric domain (CD) features (see **Fig 1**) are marked with solid circle motifs. Note that NAP19 is an unusual hybrid EEHV1B genome with an internal recombinant 24-kb EEHV1A segment cross the U60(TERex3) locus.
(TIFF)

S10 Fig Nucleotide level phylogenetic tree for the EEHV1 U71-gM locus (646-bp). Bayesian linear phylogenetic tree generated in MEGA5 by the maximum likelihood method from the same aligned nucleotide data set as in S3 Fig and with EEHV2(NAP12) used as the outgroup. The branch distance scale and some representative distance values are given. All six examples that have classic EEHV1B core chimeric domain (CD) features (see **Fig 1**) are marked with solid circle motifs. Note that NAP19 is an unusual hybrid EEHV1B genome with an internal recombinant 24-kb EEHV1A segment cross the U71-gM locus.
(TIF)

S11 Fig Nucleotide level phylogenetic tree for the EEHV1 U77(HEL) locus (952-bp). Bayesian linear phylogenetic tree generated in MEGA5 by the maximum likelihood method from the same aligned nucleotide data set as in S4 Fig and with EEHV6(NAP35) used as the outgroup. The branch distance scale and some representative distance values are given. All six examples that have classic EEHV1B core chimeric domain (CD) features (see **Fig 1**) are marked with solid circle motifs. Note that NAP19 is an unusual hybrid EEHV1B genome with an internal recombinant 24-kb EEHV1A segment cross the U77(HEL) locus.
(TIF)

S12 Fig Nucleotide level phylogenetic tree for the EEHV1 U51(vGPCR1) locus (668-bp). Bayesian linear phylogenetic tree generated in MEGA5 by the maximum likelihood method from the same aligned nucleotide data set as in S5 Fig except for omission of one sample with data less than 80% of the intact length of this locus and with EEHV6(NAP35) used as the outgroup. The branch distance scale and some representative distance values are given. All six examples that have classic EEHV1B core chimeric domain (CD) features (see **Fig 1**) are marked with solid circle motifs.
(TIF)

S13 Fig Nucleotide level phylogenetic tree for the EEHV1 E5(vGPCR5) locus (909-bp). Bayesian linear phylogenetic tree generated in MEGA5 by the maximum likelihood method from the same aligned nucleotide data set as in S6 Fig and with the EEHV1(NAP19) version used as the outgroup. The branch distance scale and some representative distance values are given. All six examples that have classic EEHV1B core chimeric domain (CD) features (see **Fig 1**) are marked with solid circle motifs.
(TIFF)

S14 Fig Nucleotide level phylogenetic tree for the EEHV1 U54(vOX2-1) locus (842-bp). Bayesian linear phylogenetic tree generated in MEGA5 by the maximum likelihood method from the same aligned nucleotide data set as in S7 Fig and with EEHV5A(EP24 = Vijay) used as the outgroup. The branch distance scale and some representative distance values are given. All six examples that have classic EEHV1B core chimeric domain (CD) features (see **Fig 1**) are marked with solid circle motifs.
(TIFF)

S15 Fig Selected protein level phylogenetic tree for the EEHV1 U51(vGPCR1) PCR locus (222-aa). Bayesian linear phylogenetic tree generated from translated amino acid data in MEGA5 by the maximum likelihood method from the matching aligned dataset as in the S5 and S12 Figs and with EEHV6(NAP35) used as the outgroup. The branch distance scale and some representative distance values are given. All six examples that have classic EEHV1B core chimeric domain (CD) features (see **Fig 1**) are marked with solid circle motifs.
(TIF)

S16 Fig Selected protein level phylogenetic tree for the EEHV1 E5(vGPCR5) PCR locus (291-aa). Bayesian linear phylogenetic tree generated from translated amino acid data in MEGA5 by the maximum likelihood method from the matching aligned dataset as in the S6 and S13 Figs and with the EEHV1(NAP19) version used as the outgroup. The branch distance scale and some representative distance values are given. All six examples that have classic EEHV1B core chimeric domain (CD) features (see **Fig 1**) are marked with solid circle motifs.
(TIFF)

S17 Fig Selected protein level phylogenetic tree for the EEHV1 E54(vOX2-1) PCR locus (280-aa). Bayesian linear phylogenetic tree generated from translated amino acid data in MEGA5 by the maximum likelihood method from the matching dataset as in the S7 and S14 Figs and with EEHV5A(EP24 = Vijay) used as the outgroup. The branch distance scale and some representative distance values are given. All six examples that have classic EEHV1B core chimeric domain (CD) features (see **Fig 1**) are marked with solid circle motifs.
(TIFF)

## References

[1] Ossent P, Guscetti F, Metzler AE, Lang EM, Rübel A, Hauser B. Acute and fatal herpesvirus infection in a young Asian elephant (Elephas maximus). Vet Pathol. 1990;27:131–3. 10.1177/030098589002700212 2161138

[2] Richman LK, Montali RJ, Garber RL, Kennedy MA, Lehnhardt J, Hildebrandt T, et al Novel endotheliotropic herpesviruses fatal for Asian and African elephants. Science. 1999a;283:1171–6. 10024244 10.1126/science.283.5405.1171

[3] Richman LK, Montali RJ, Cambre RC, Schmitt D, Hardy D, Hildbrandt T, et al Clinical and pathological findings of a newly recognized disease of elephants caused by endotheliotropic herpesviruses. J Wildl Dis. 2000a;36:1–12. 10682740 10.7589/0090-3558-36.1.1

[4] Hayward GS. Conservation: clarifying the risk from herpesvirus to captive Asian elephants. Vet Rec. 2012;170:202–3. 10.1136/vr.e1212 22368209 PMC3587150

[5] Long SY, Latimer E, Hayward GS. Review of Elephant Endotheliotropic Herpesviruses and Acute Hemorrhagic Disease. ILAR Journal. 2016;56:283–96. 10.1093/ilar/ilv041 26912715 PMC4765743

[6] Kendall R, Howard L, Masters N, Grant R. The Impact of Elephant Endotheliotropic Herpesvirus on the Captive Asian Elephant (Elephas Maximus) Population of the United Kingdom and Ireland (1995–2013). J Zoo Wildl Med. 2016;47:405–18. 10.1638/2015-0217.1 27468010

[7] Wiedner E, Howard L, Isaza R. Treatment of elephant endotheliotropic herpes virus (EEHV). In: Fowler ME, Miller RE, editors. Zoo and Wild Animal Medicine. 7th ed2012. p. 537–43.

[8] Schmitt DL, Hardy DA, Montali RJ, Richman LK, Lindsay WA, Isaza R, et al Use of famciclovir for the treatment of endotheliotrophic herpesvirus infections in Asian elephants (Elephas maximus). J Zoo Wildl Med. 2000;31:518–22. 10.1638/1042-7260(2000)031[0518:UOFFTT]2.0.CO;2 11428400

[9] Garner MM, Helmick K, Ochsenreiter J, Richman LK, Latimer E, Wise AG, et al Clinico-pathologic features of fatal disease attributed to new variants of endotheliotropic herpesviruses in two Asian elephants (Elephas maximus). Vet Pathol. 2009;46:97–104. 10.1354/vp.46-1-97 19112123 PMC3572918

[10] Latimer E, Zong J-C, Heaggans SY, Richman LK, Hayward GS. Detection and evaluation of novel herpesviruses in routine and pathological samples from Asian and African elephants: identification of two new probosciviruses (EEHV5 and EEHV6) and two new gammaherpesviruses (EGHV3B and EGHV5). Vet Microbiol. 2011;147:28–41. 10.1016/j.vetmic.2010.05.042 20579821 PMC2976818

[11] Atkins L, Zong J-C, Tan J, Mejia A, Heaggans SY, Nofs SA, et al EEHV-5, a newly recognized elephant herpesvirus associated with clinical and subclinical infections in captive asian elephants (Elephas maximus). J Zoo Wildl Med. 2013;44:136–43. 10.1638/1042-7260-44.1.136 23505714 PMC3746547

[12] Fuery A, Browning GR, Tan J, Long SY, Hayward GS, Cox SK, et al Clinical infection of captive Asian elephants (Elephas Maximus) with elephant endotheliotropic herpesvirus 4. J Zoo Wildl Med. 2016;47:311–8. 10.1638/2015-0072.1 27010293

[13] Zong J-C, Latimer EM, Long SY, Richman LK, Heaggans SY, Hayward GS. Comparative genome analysis of four elephant endotheliotropic herpesviruses, EEHV3, EEHV4, EEHV5, and EEHV6, from cases of hemorrhagic disease or viremia. J Virol. 2014;88:13547–69. 10.1128/JVI.01675-14 25231309 PMC4248975

[14] Richman LK, Zong J-C, Latimer E, Lock J, Fleischer RC, Heaggans SY, et al Elephant endotheliotropic herpesviruses EEHV1A, EEHV1B, and EEHV2 from cases of hemorrhagic disease are highly diverged from other mammalian herpesviruses and may form a new subfamily. J Virol. 2014;88:13523–46. 10.1128/JVI.01673-14 25231303 PMC4248956

[15] Zong J-C, Heaggans SY, Long SY, Latimer EM, Nofs SA, Bronson E, et al Detection of quiescent infections with multiple elephant endotheliotropic herpesviruses (EEHVs), including EEHV2, EEHV3, EEHV6 and EEHV7, within lymphoid lung nodules or lung and spleen tissue samples from five asymptomatic adult african elephants. J Virol. 2015;90:3028–43. 10.1128/JVI.02936-15 26719245 PMC4810643

[16] Stanton JJ, Nofs SA, Peng R, Hayward GS, Ling PD. Development and validation of quantitative real-time polymerase chain reaction assays to detect elephant endotheliotropic herpesviruses-2, 3, 4, 5, and 6. J Virol Meths. 2012;186:73–7. 10.1016/j.jviromet.2012.07.024 PMC3506426 22842286

[17] Stanton JJ, Zong J-C, Eng C, Howard L, Flanagan J, Stevens S, et al Kinetics of viral loads and genotypic analysis of elephant endotheliotropic herpesvirus-1 infection in captive Asian elephants (Elephas maximus). J Zoo Wildl Med. 2013;44:42–54. 10.1638/1042-7260-44.1.42 23505702 PMC3746492

[18] Pursell T, Tan J, Peng R, Ling PD. Generation and validation of new quantitative real time PCR assays to detect elephant endotheliotropic herpesviruses 1A, 1B, and 4. J Virol Methods. 2016;237:138–42. 10.1016/j.jviromet.2016.08.010 27542531

[19] Stanton JJ, Zong J-C, Latimer E, Tan J, Herron A, Hayward GS, et al Detection of pathogenic elephant endotheliotropic herpesvirus in routine trunk washes from healthy adult Asian elephants (Elephas maximus) by use of a real-time quantitative polymerase chain reaction assay. Am J Vet Res. 2010;71:925–33. 10.2460/ajvr.71.8.925 20673092 PMC3725808

[20] Zachariah A, Zong J-C, Long SY, Latimer EM, Heaggans SY, Richman LK, et al Fatal herpesvirus (EEHV) hemorrhagic disease in wild and orphan Asian elephants in Southern India. J Wildl Dis. 2013;49:383–91. 10.7589/2012-07-193 PMC3707512 23568914

[21] Sripiboon S, Tankaew P, Lungka G, Thitaram C. The occurrence of elephant endotheliotropic herpesvirus in captive Asian elephants (Elephas maximus): first case of EEHV4 in Asia. J Zoo Wildl Med. 2013;44:100–4. 10.1638/1042-7260-44.1.100 23505709

[22] Bouchard B, Xaymountry B, Thongtip N, Lertwatcharasarakul P, Wajjwalku W. First reported case of elephant endotheliotropic herpes virus infection in Laos. J Zoo Wildl Med. 2014;45:704–7. 10.1638/2013-0264R1.1 25314848

[23] Sripiboon S, Jackson B, Ditcham W, Holyoake C, Robertson I, Thitaram C, et al Molecular characterisation and genetic variation of Elephant Endotheliotropic Herpesvirus infection in captive young Asian elephants in Thailand. Infect Genet Evol. 2016;44:487–94. 10.1016/j.meegid.2016.08.004 27503594

[24] Reid CE, Hildebrandt TB, Marx N, Hunt M, Thy N, Reynes JM, et al Endotheliotropic elephant herpes virus (EEHV) infection. The first PCR-confirmed fatal case in Asia. Vet Q. 2006;28:61–4. 10.1080/01652176.2006.9695209 16841568

[25] Ling PD, Reid JG, Qin X, Muzny DM, Gibbs R, Petrosino J, et al Complete genome sequence of elephant endotheliotropic herpesvirus 1A. Genome Announc. 2013;1:e0010613 10.1128/genomeA.00106-13 23580705 PMC3624679

[26] Wilkie GS, Davison AJ, Watson M, Kerr K, Sanderson S, Bouts T, et al Complete genome sequences of elephant endotheliotropic herpesviruses 1A and 1B determined directly from fatal cases. J Virol. 2013;87:6700–12. 10.1128/JVI.00655-13 23552421 PMC3676107

[27] Wilkie GS, Davison AJ, Kerr K, Stidworthy MF, Redrobe S, Steinbach F, et al First fatality associated with elephant endotheliotropic herpesvirus 5 in an asian elephant: pathological findings and complete viral genome sequence. Scientific reports. 2014;4:6299 10.1038/srep06299 25199796 PMC5385831

[28] Wilkie NM, Davison A, Chartrand P, Stow ND, Preston VG, Timbury MC. Recombination in herpes simplex virus: mapping of mutations and analysis of intertypic recombinants. Cold Spring Harb Symp Quant Biol. 1979;43 Pt 2:827–40. 226325 10.1101/sqb.1979.043.01.089

[29] Ling PD, Long SY, Fuery A, Peng R, Heaggans SY, Qin X, et al Complete genome sequence of elephant endotheliotropic herpesvirus 4 (EEHV4) the first example of a GC-rich branch proboscivirus. mSphere. 2016a;1:e00081–15. 27340695 10.1128/mSphere.00081-15 PMC4911795

[30] Ling PD, Long SY, Heaggans SY, Qin X, Hayward GS. Comparison of the gene coding contents and other unusual features of the GC-rich and AT-rich branch probosciviruses. mSphere. 2016b;1:e00091–16. 27340696 10.1128/mSphere.00091-16 PMC4911796

[31] Pellett PE. Trunkloads of viruses. Journal of virology. 2014;88(23):13520–2. 10.1128/JVI.02359-14 25231304 PMC4248977

[32] Zong J-C, Latimer EM, Heaggans SY, Richman LK, Hayward GS. Pathogenesis and molecular epidemiology of fatal elephant endotheliotropic disease associated with the expanding proboscivirus genus of the betaherpesvirinae. Proc Internat Elephant Cons and Res Symp; Florida 2008 pp.23–35.

[33] Lertwatcharasarakul P, Sanyathitiseree P, Thongtip N, Charoenphan P, Boonyasart B, Maneewan N, et al Genetic Variant of Elephant Endotheliotropic Herpesvirus Detected from Captive Asian Elephants (Elephas maximus) in Thailand from 2007 to 2013. Thai J Vet Med. 2015;45:73.

[34] McGeoch DJ, Rixon FJ, Davison AJ. Topics in herpesvirus genomics and evolution. Virus research. 2006;117:90–104. 10.1016/j.virusres.2006.01.002 16490275

[35] Vidya TN, Fernando P, Melnick DJ, Sukumar R. Population differentiation within and among Asian elephant (Elephas maximus) populations in southern India. Heredity (Edinb). 2005;94:71–80. 15454948 10.1038/sj.hdy.6800568

[36] Vidya TNC, Fernando P, Melnick DJ, Sukumar R. Population genetic structure and conservation of Asian elephants (Elephas macimus) across India. Animal Conservation. 2005;8:377–88.

