# Supplementary figures and images for: Extended genotypic evaluation and comparison of twenty-two cases of lethal EEHV1 hemorrhagic disease in wild and captive Asian elephants in India
Source: PLoS One. 2018 Aug 22;13(8):e0202438. doi: 10.1371/journal.pone.0202438 (PMC6105008; doi:10.1371/journal.pone.0202438)

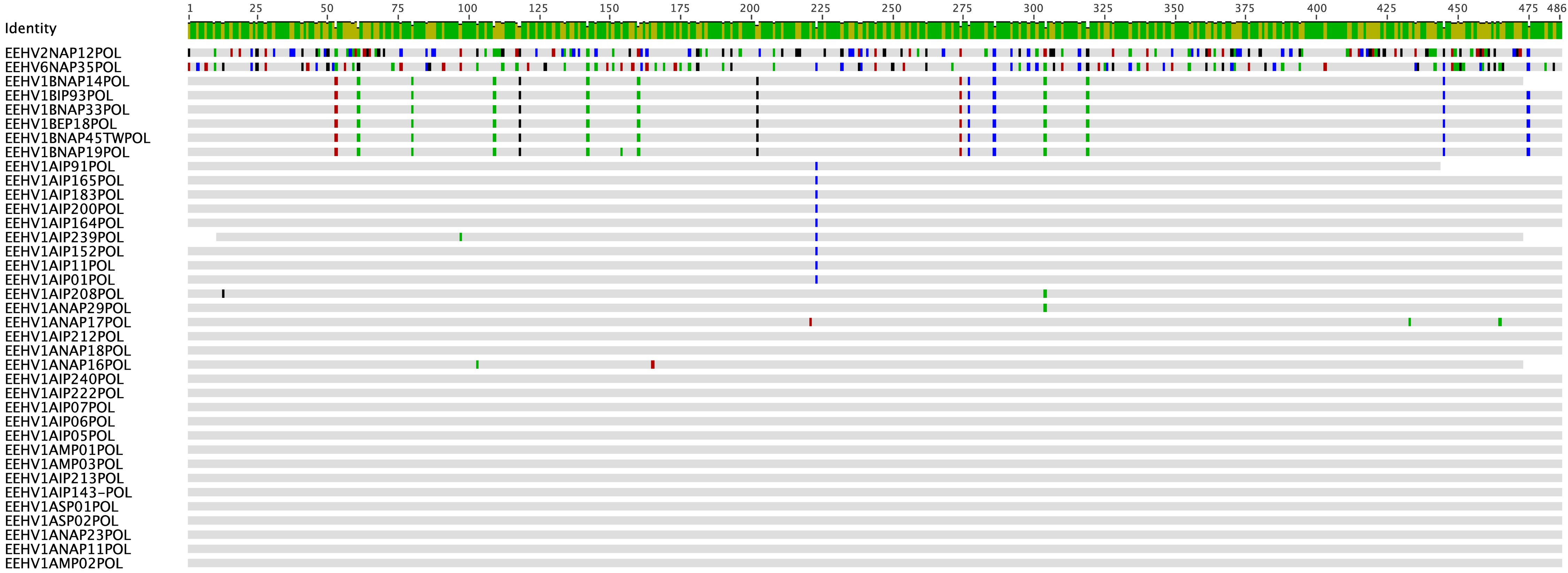

Supplement: S1 Fig — As for Fig 2 except for also including data for the two Sumatran cases (SP#) and the use of both the prototype EEHV2(NAP12) and EEHV6(NAP35) genome data for comparison. (TIF) [file pone.0202438.s001.tif]

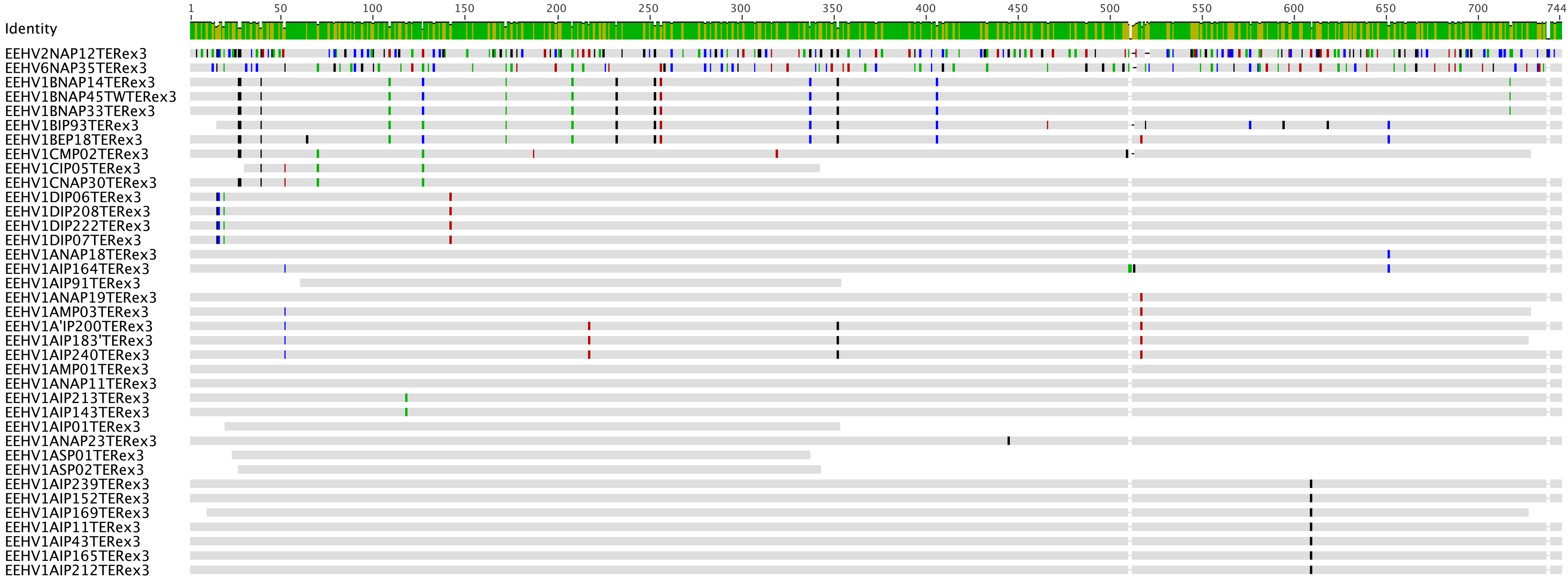

Supplement: S2 Fig — As for Fig 2 except for also including data for the two Sumatran cases (SP#) and the use of both the prototype EEHV2(NAP12) and EEHV6(NAP35) genome data for comparison. (TIF) [file pone.0202438.s002.tif]

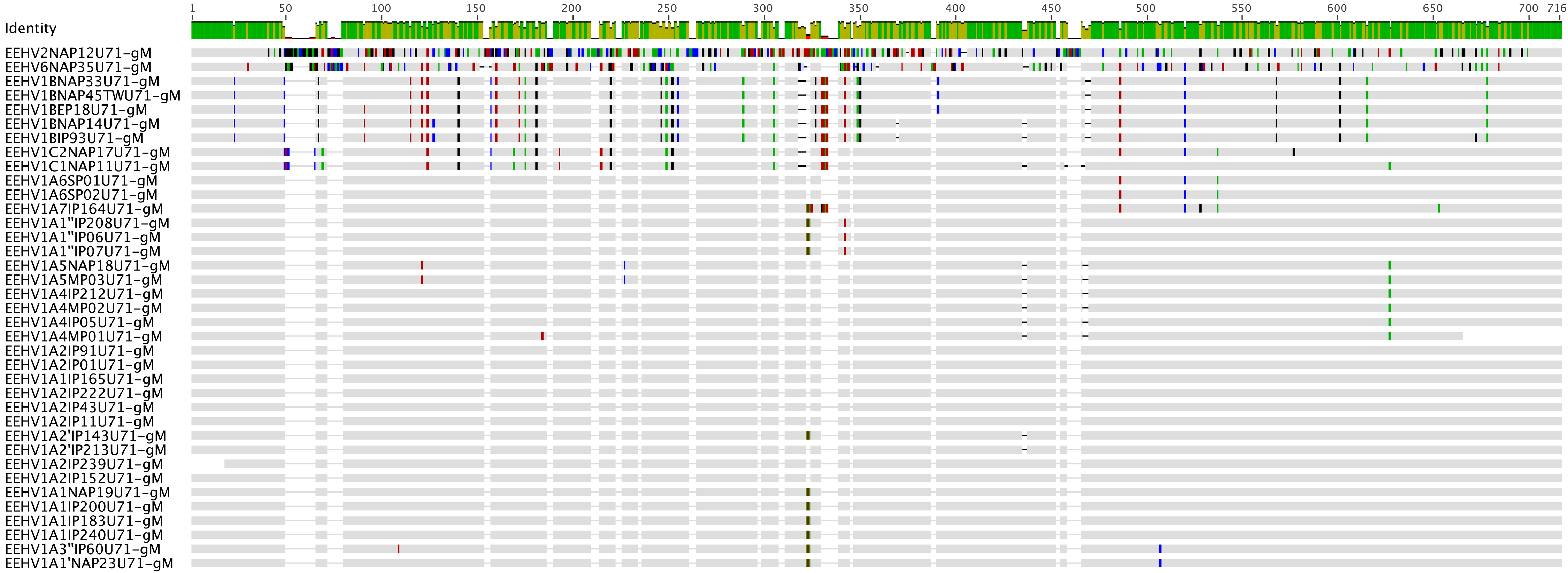

Supplement: S3 Fig — As for Fig 2 except for also including data for the two Sumatran cases (SP#) and the use of both the prototype EEHV2(NAP12) and EEHV6(NAP35) genome data for comparison. (TIF) [file pone.0202438.s003.tif]

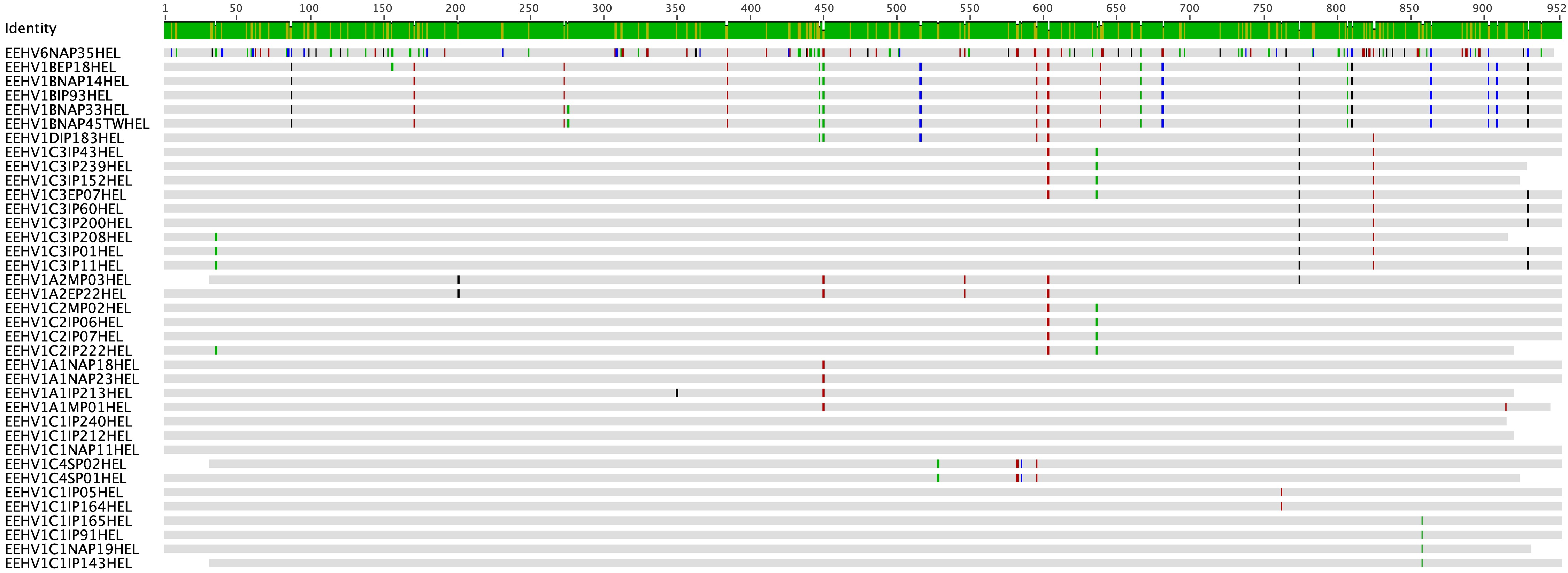

Supplement: S4 Fig — As for Fig 2 except for also including data for the two Sumatran cases (SP#) and the use of the EEHV6(NAP35) genome data rather than EEHV2(NAP12) for comparison. (TIF) [file pone.0202438.s004.tif]

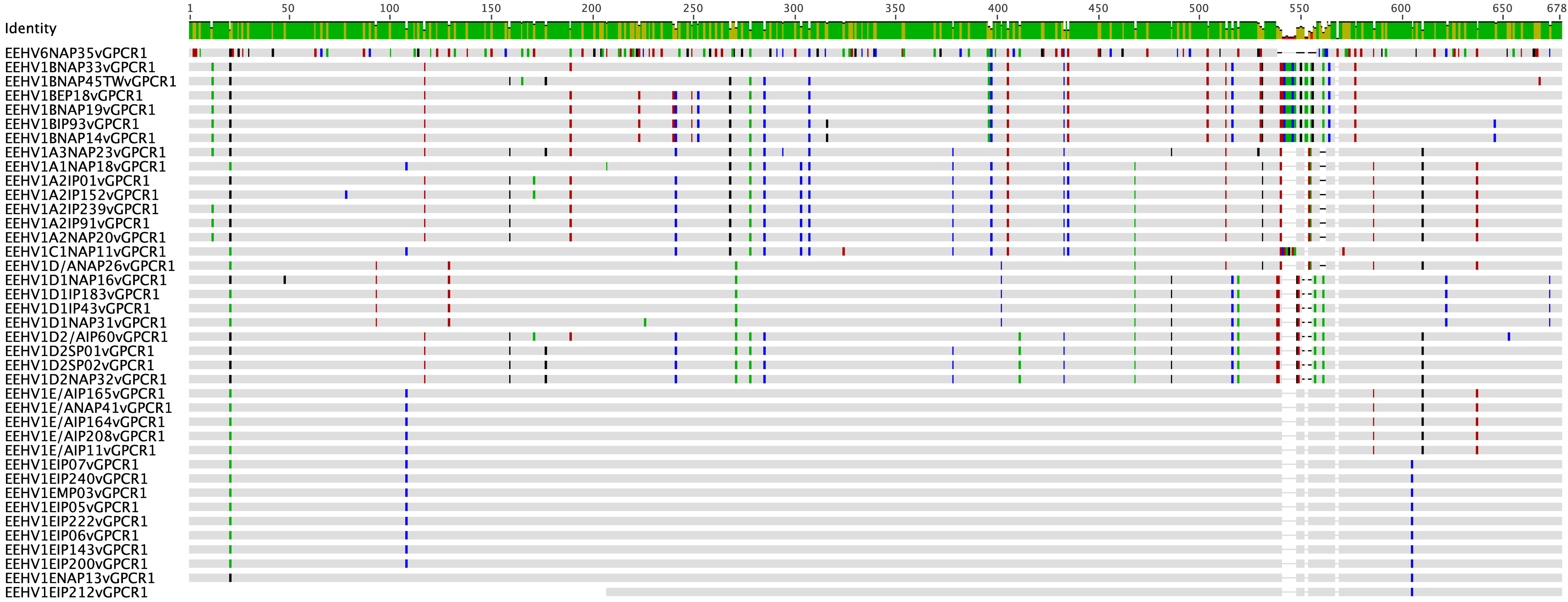

Supplement: S5 Fig — As for Fig 2 except for also including data for the two Sumatran cases (SP#) and the use of the prototype EEHV6(NAP35) genome data rather than EEHV2(NAP2) for comparison. (TIF) [file pone.0202438.s005.tif]

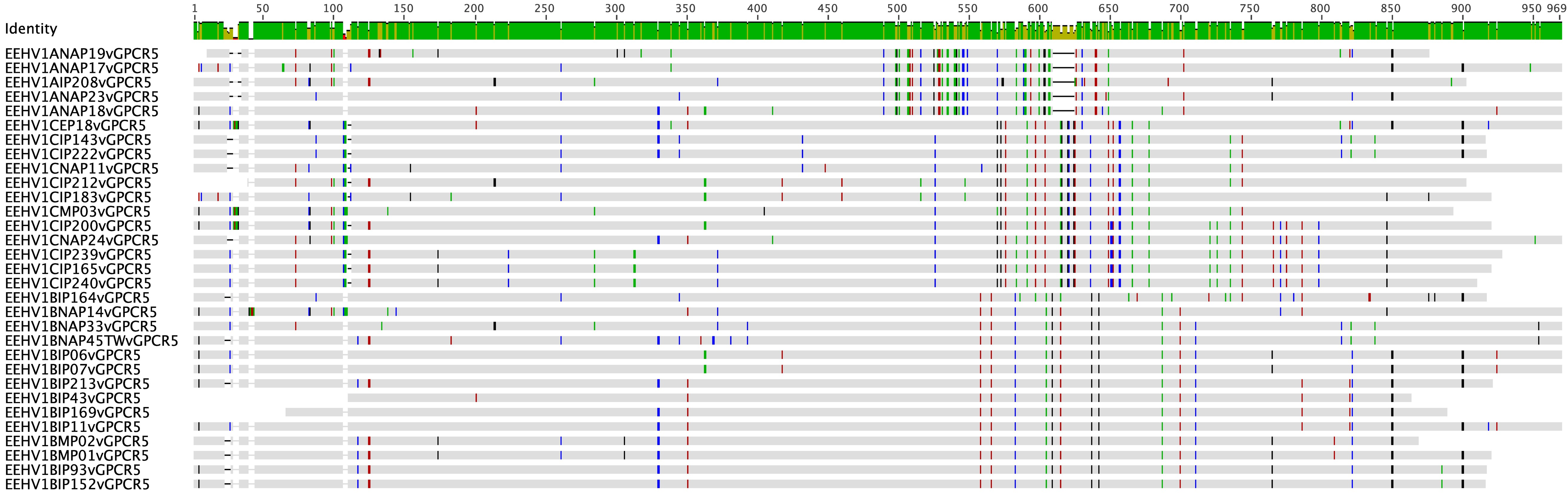

Supplement: S6 Fig — As for Fig 2 except that no equivalent data is available for comparison from either EEHV2 or EEHV6. (TIF) [file pone.0202438.s006.tif]

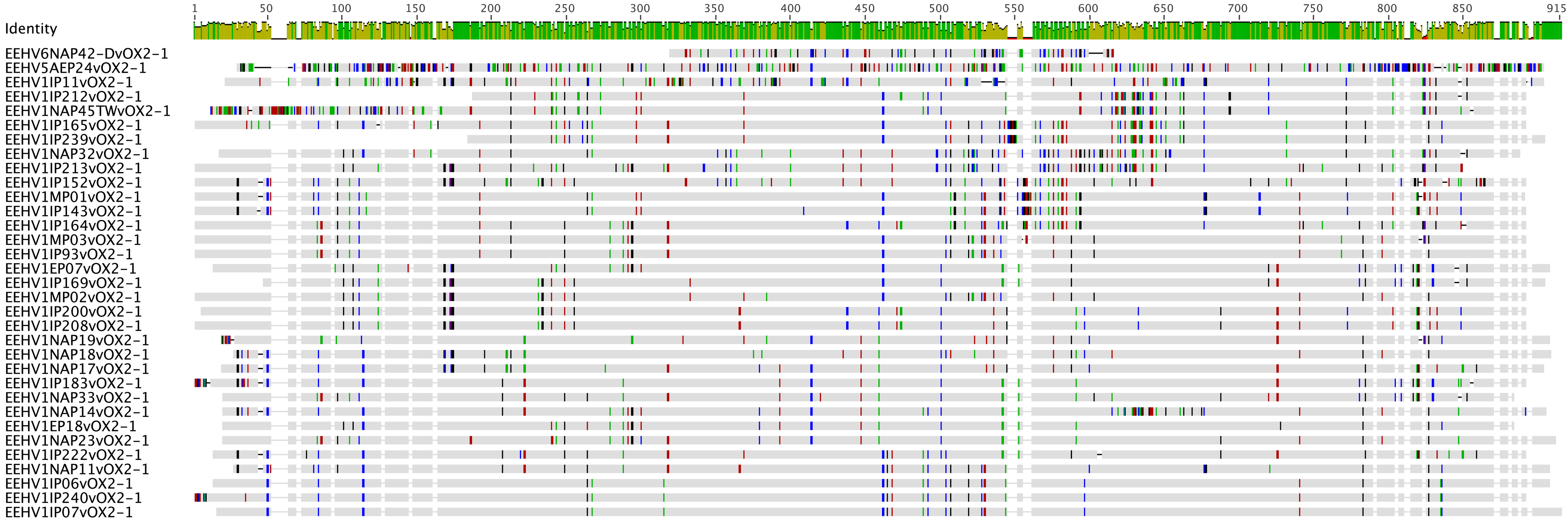

Supplement: S7 Fig — As for Fig 2 except for also including partial data for EEHV6(NAP42D) and intact matching data for the EEHV5A(EP24 = Vijay) genome for comparison. (TIF) [file pone.0202438.s007.tif]

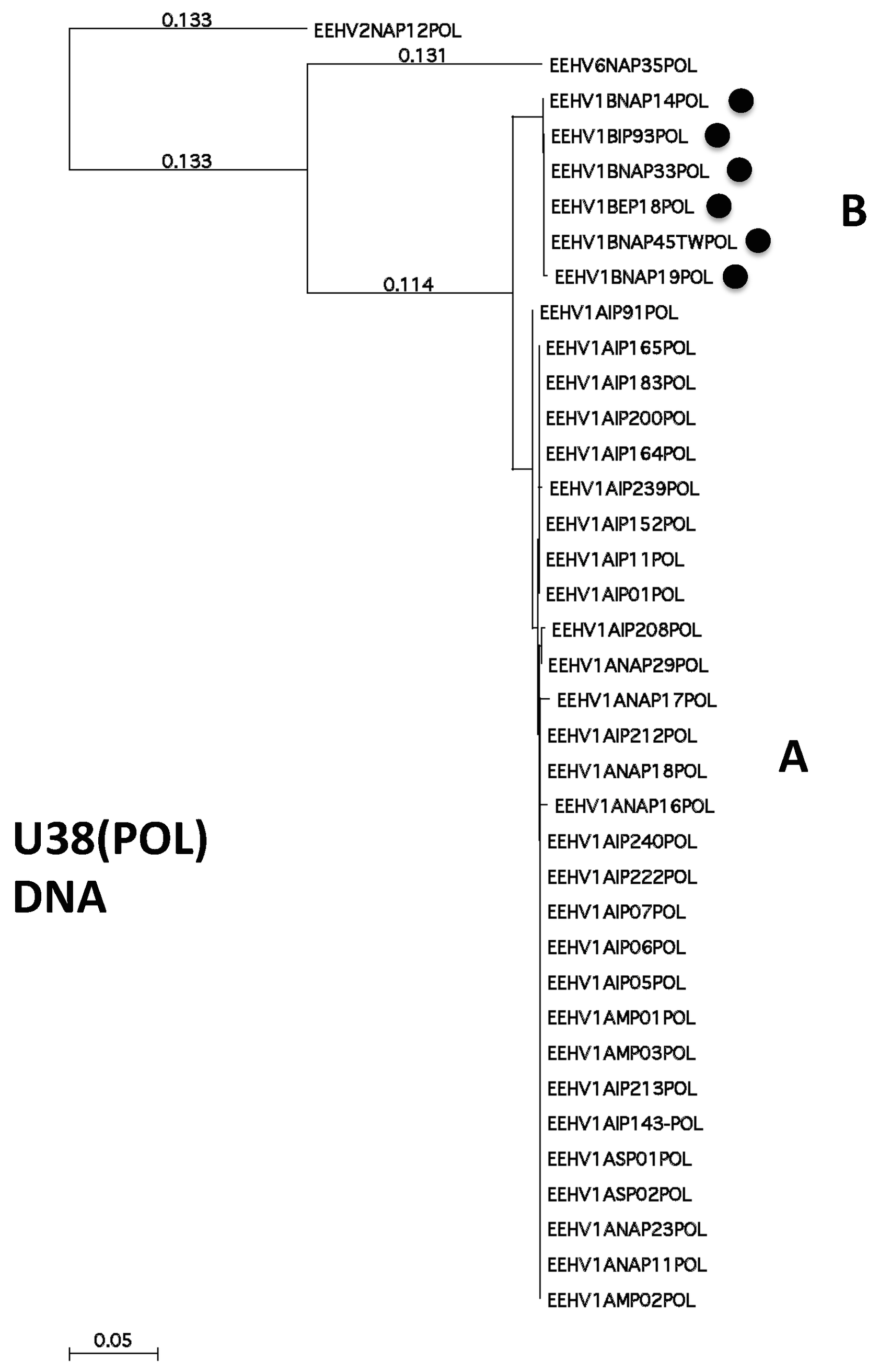

Supplement: S8 Fig — Bayesian linear phylogenetic tree generated in MEGA5 by the maximum likelihood method from the same aligned nucleotide data set as in S1 Fig and with EEHV2(NAP12) used as the outgroup. The branch distance scale and some representative distance values are given. All six examples that have classic EEHV1B core chimeric domain (CD) features (see Fig 1) are marked with solid circle motifs. (TIF) [file pone.0202438.s008.tif]

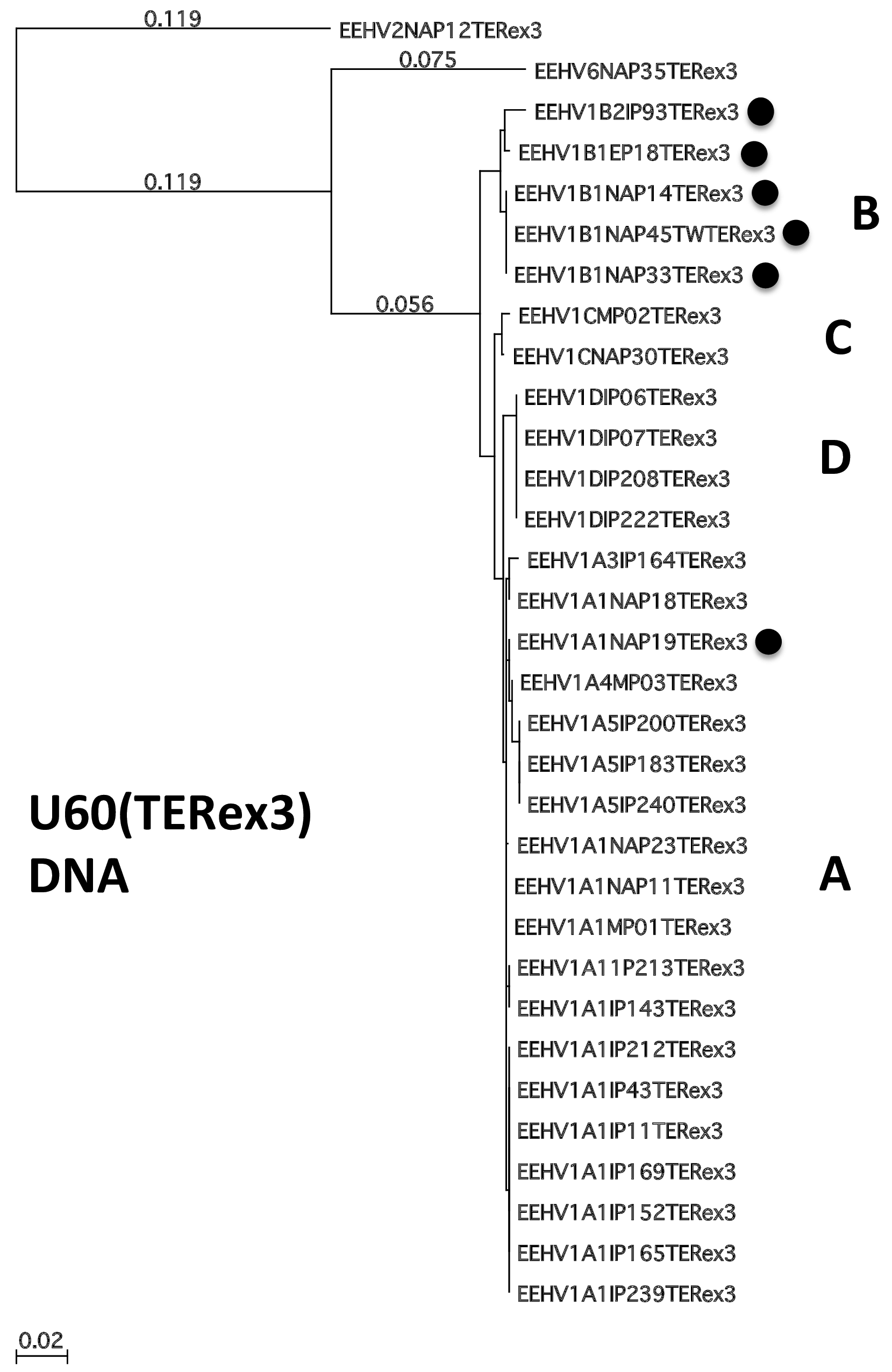

Supplement: S9 Fig — Bayesian linear phylogenetic tree generated in MEGA5 by the maximum likelihood method from the same aligned nucleotide data set as in S2 Fig and with EEHV2(NAP12) used as the outgroup, except for the omission of five samples with data less than 80% of the length of the intact locus. The branch distance scale and some representative distance values are given. All six examples that have classic EEHV1B core chimeric domain (CD) features (see Fig 1) are marked with solid circle motifs. Note that NAP19 is an unusual hybrid EEHV1B genome with an internal recombinant 24-kb EEHV1A segment cross the U60(TERex3) locus. (TIFF) [file pone.0202438.s009.tiff]

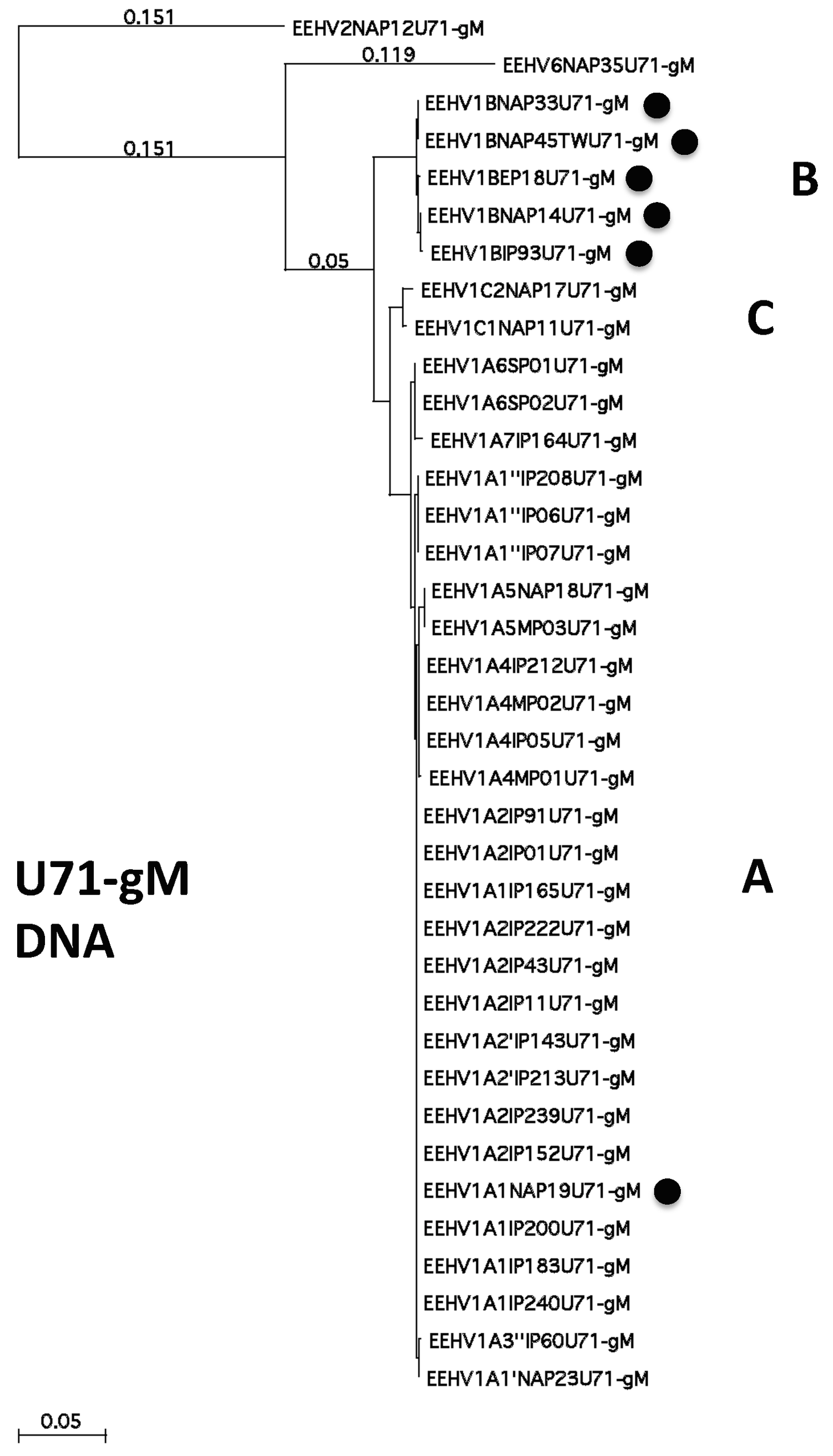

Supplement: S10 Fig — Bayesian linear phylogenetic tree generated in MEGA5 by the maximum likelihood method from the same aligned nucleotide data set as in S3 Fig and with EEHV2(NAP12) used as the outgroup. The branch distance scale and some representative distance values are given. All six examples that have classic EEHV1B core chimeric domain (CD) features (see Fig 1) are marked with solid circle motifs. Note that NAP19 is an unusual hybrid EEHV1B genome with an internal recombinant 24-kb EEHV1A segment cross the U71-gM locus. (TIF) [file pone.0202438.s010.tif]

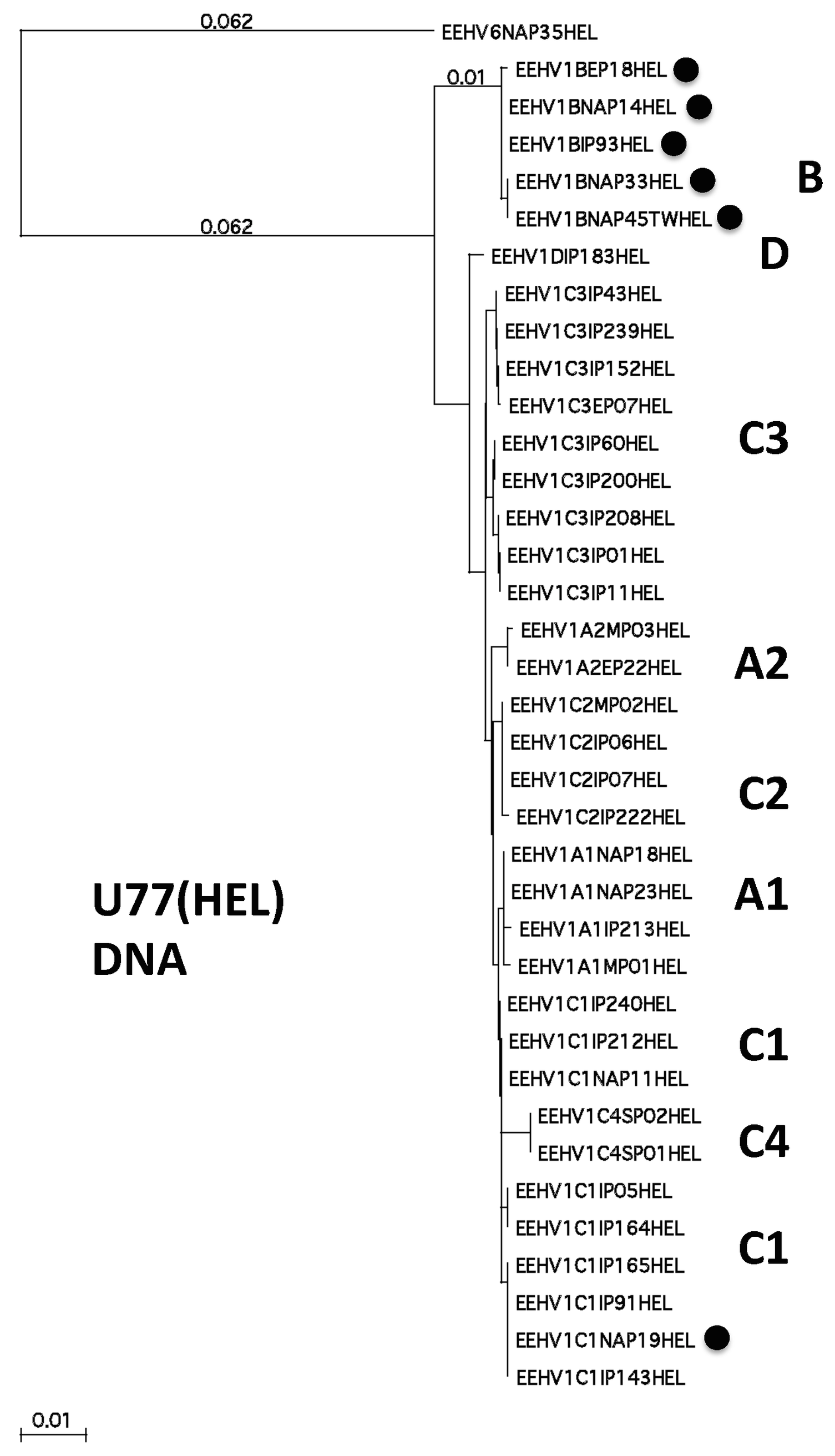

Supplement: S11 Fig — Bayesian linear phylogenetic tree generated in MEGA5 by the maximum likelihood method from the same aligned nucleotide data set as in S4 Fig and with EEHV6(NAP35) used as the outgroup. The branch distance scale and some representative distance values are given. All six examples that have classic EEHV1B core chimeric domain (CD) features (see Fig 1) are marked with solid circle motifs. Note that NAP19 is an unusual hybrid EEHV1B genome with an internal recombinant 24-kb EEHV1A segment cross the U77(HEL) locus. (TIF) [file pone.0202438.s011.tif]

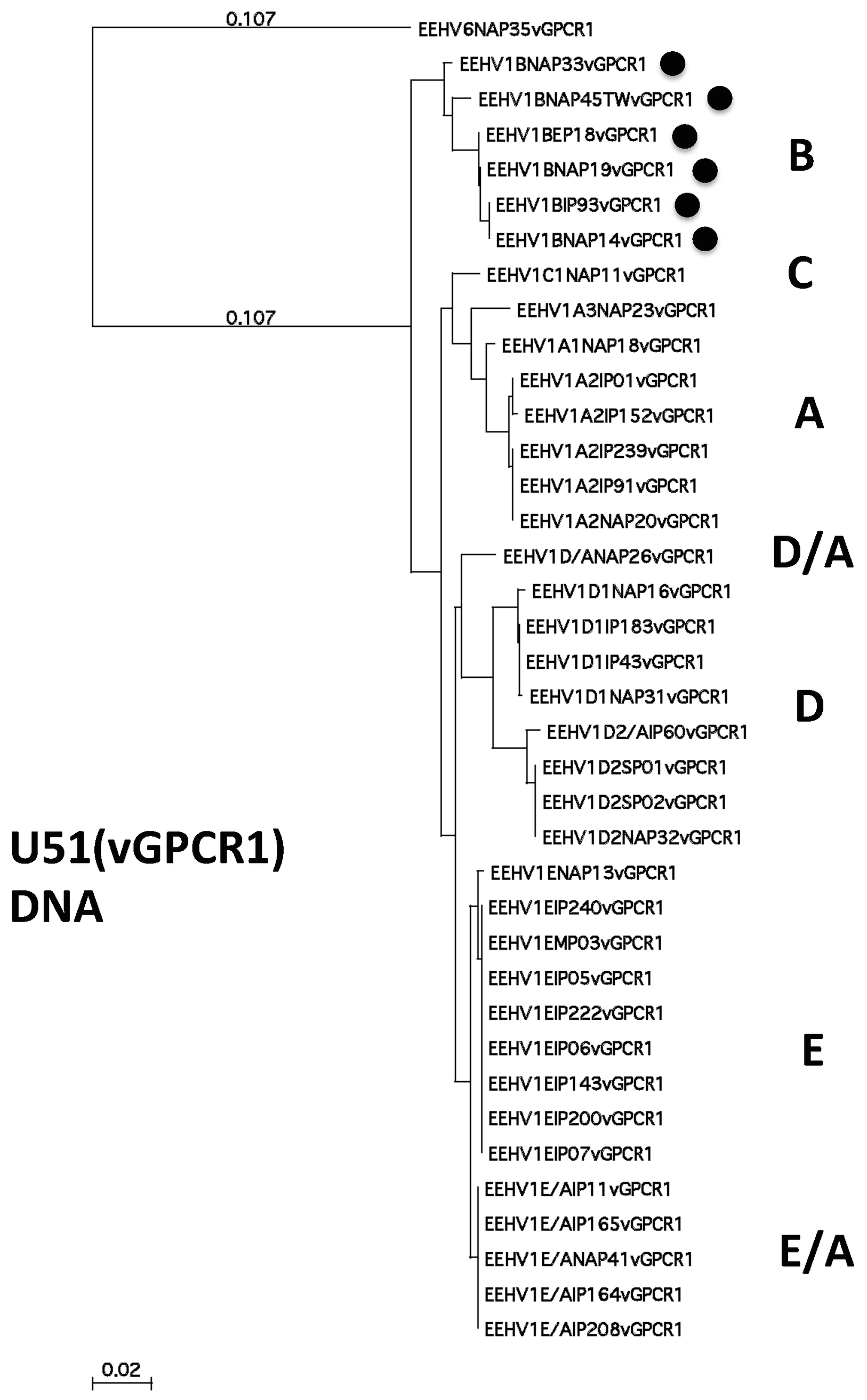

Supplement: S12 Fig — Bayesian linear phylogenetic tree generated in MEGA5 by the maximum likelihood method from the same aligned nucleotide data set as in S5 Fig except for omission of one sample with data less than 80% of the intact length of this locus and with EEHV6(NAP35) used as the outgroup. The branch distance scale and some representative distance values are given. All six examples that have classic EEHV1B core chimeric domain (CD) features (see Fig 1) are marked with solid circle motifs. (TIF) [file pone.0202438.s012.tif]

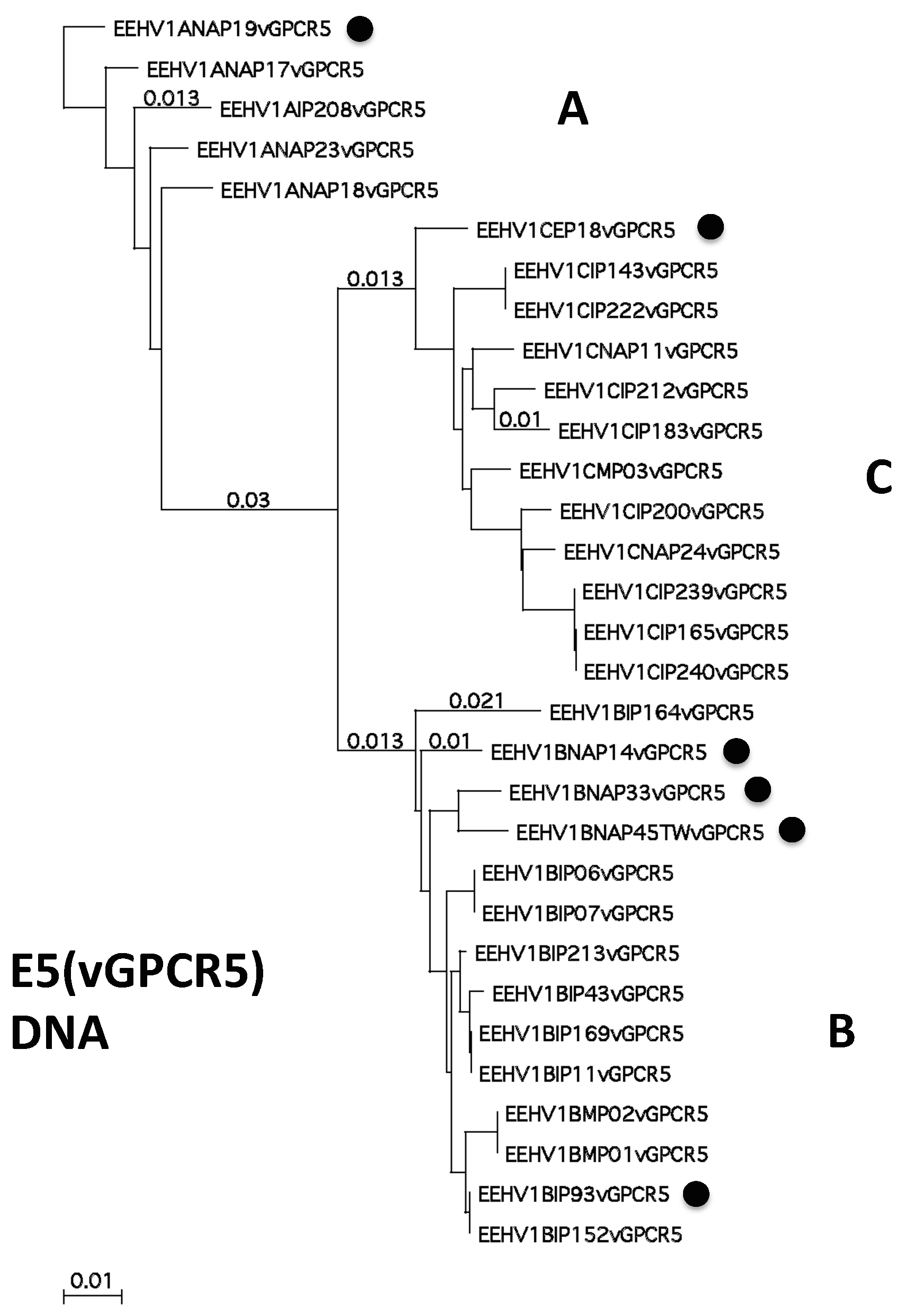

Supplement: S13 Fig — Bayesian linear phylogenetic tree generated in MEGA5 by the maximum likelihood method from the same aligned nucleotide data set as in S6 Fig and with the EEHV1(NAP19) version used as the outgroup. The branch distance scale and some representative distance values are given. All six examples that have classic EEHV1B core chimeric domain (CD) features (see Fig 1) are marked with solid circle motifs. (TIFF) [file pone.0202438.s013.tiff]

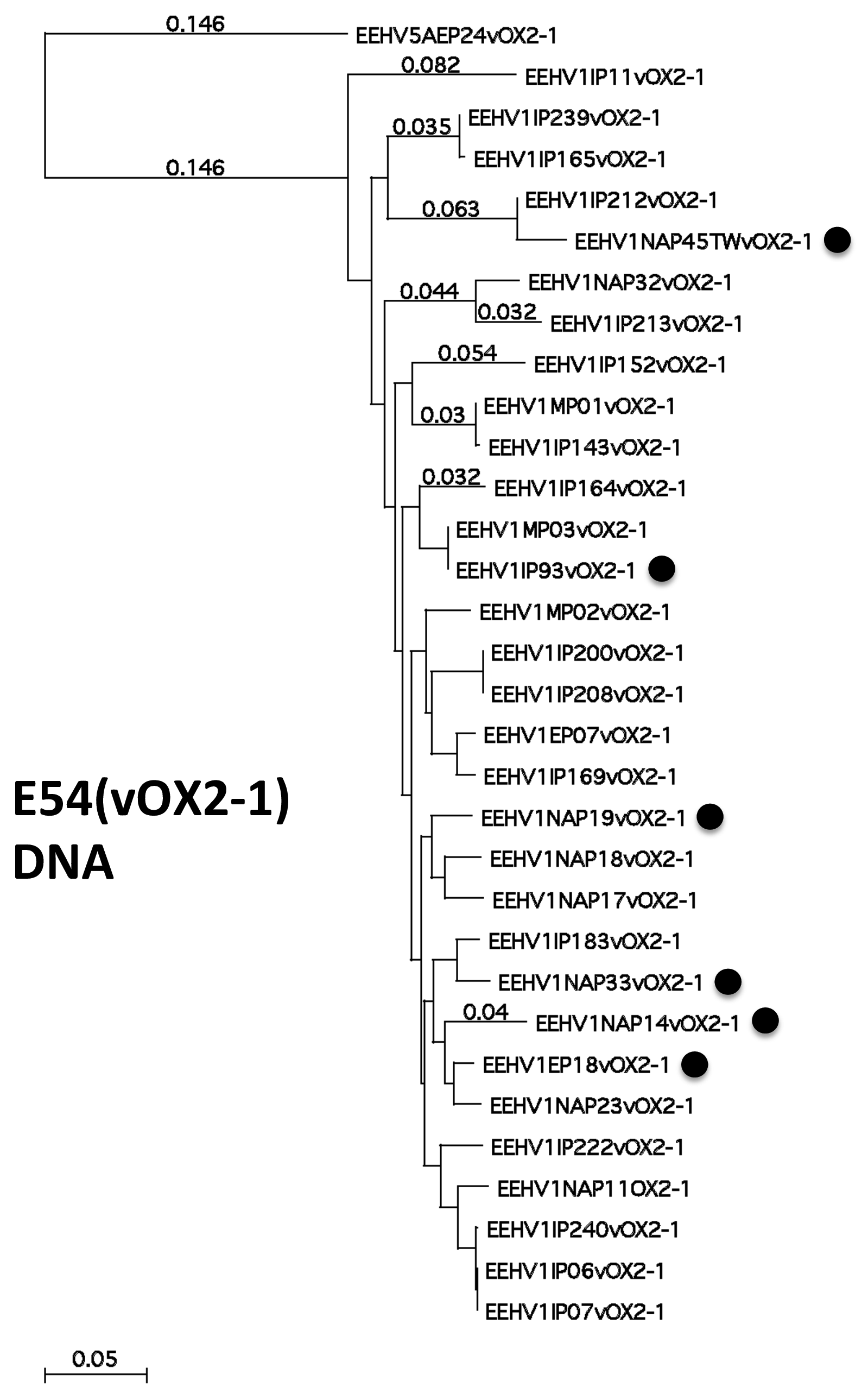

Supplement: S14 Fig — Bayesian linear phylogenetic tree generated in MEGA5 by the maximum likelihood method from the same aligned nucleotide data set as in S7 Fig and with EEHV5A(EP24 = Vijay) used as the outgroup. The branch distance scale and some representative distance values are given. All six examples that have classic EEHV1B core chimeric domain (CD) features (see Fig 1) are marked with solid circle motifs. (TIFF) [file pone.0202438.s014.tiff]

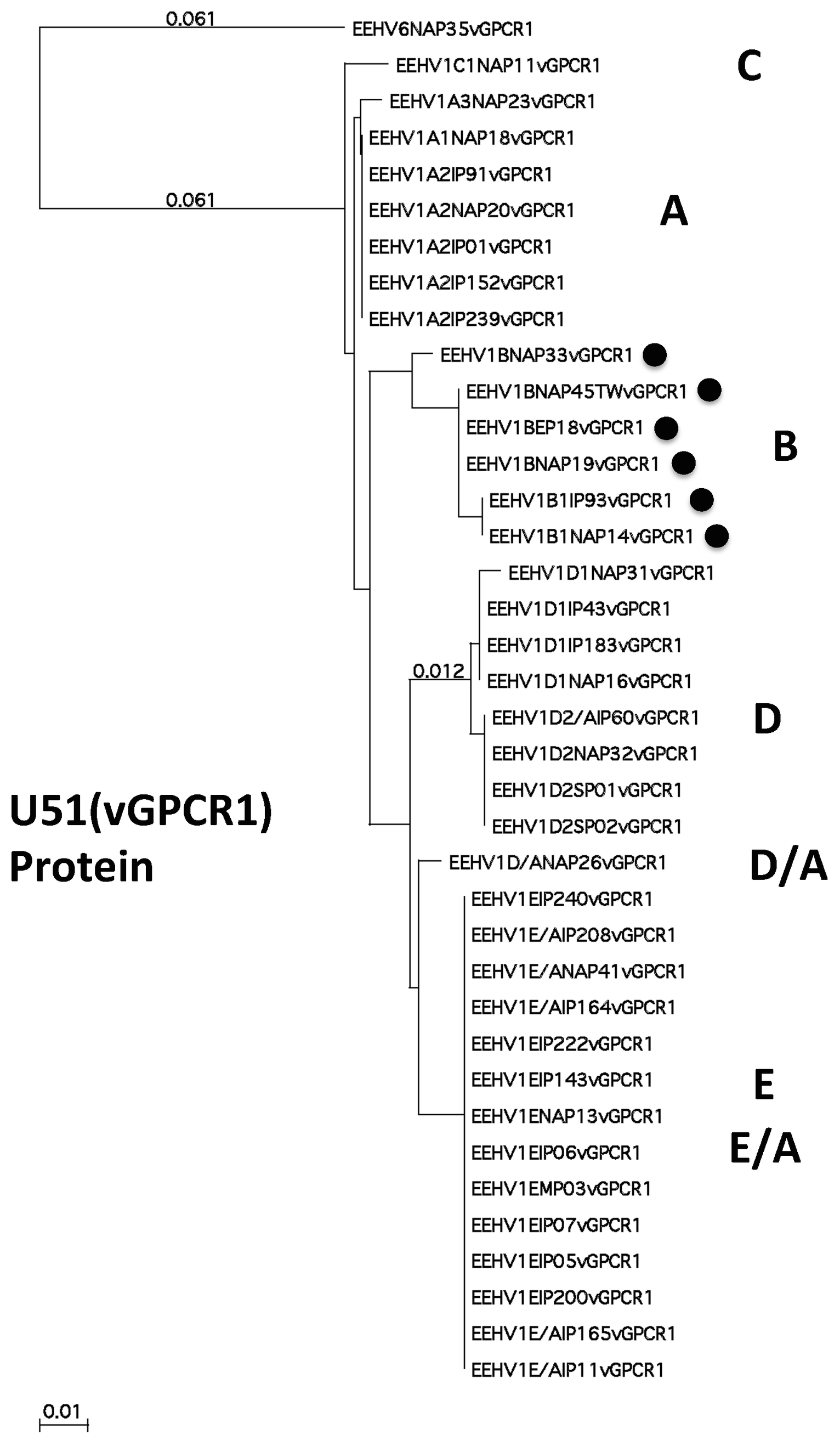

Supplement: S15 Fig — Bayesian linear phylogenetic tree generated from translated amino acid data in MEGA5 by the maximum likelihood method from the matching aligned dataset as in the S5 and S12 Figs and with EEHV6(NAP35) used as the outgroup. The branch distance scale and some representative distance values are given. All six examples that have classic EEHV1B core chimeric domain (CD) features (see Fig 1) are marked with solid circle motifs. (TIF) [file pone.0202438.s015.tif]

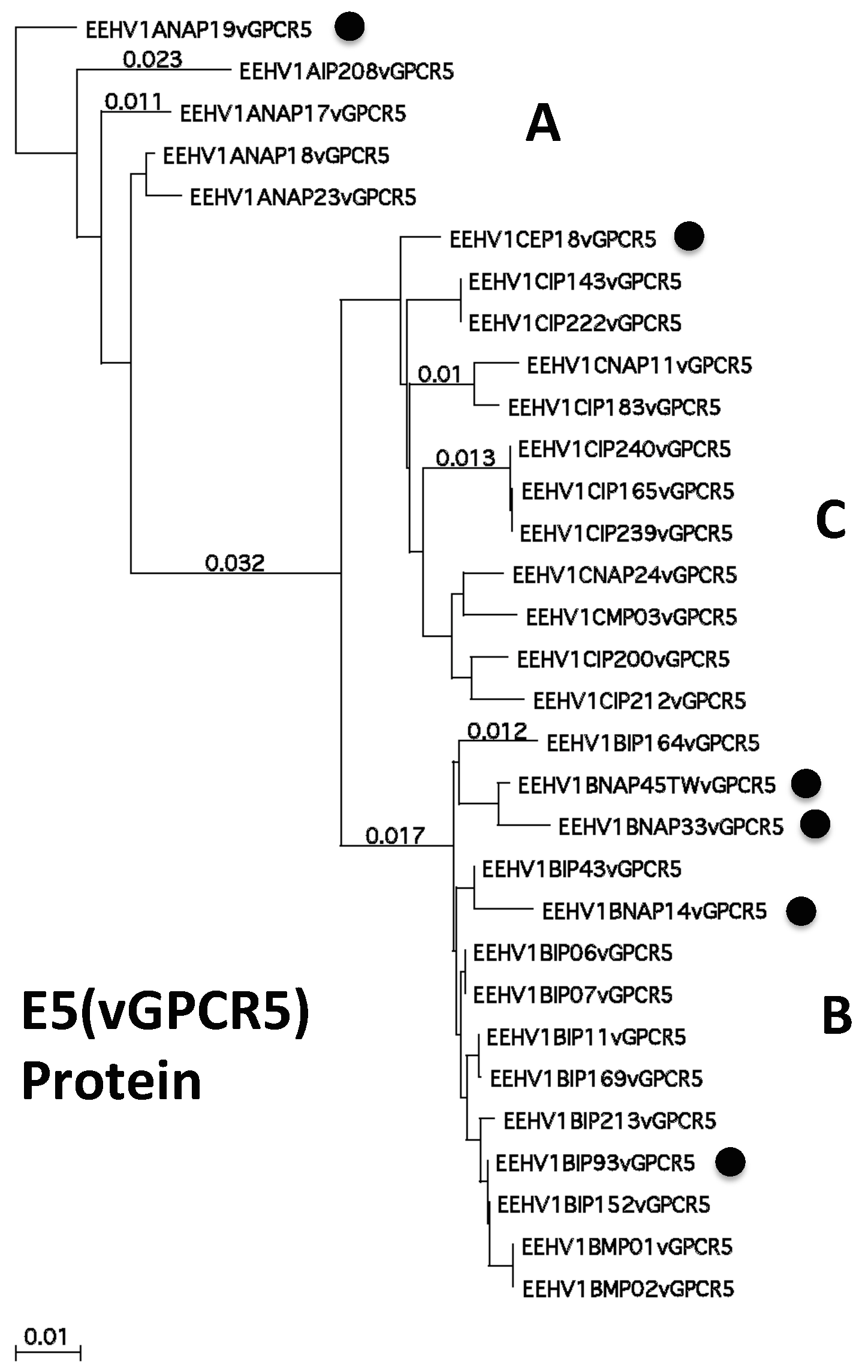

Supplement: S16 Fig — Bayesian linear phylogenetic tree generated from translated amino acid data in MEGA5 by the maximum likelihood method from the matching aligned dataset as in the S6 and S13 Figs and with the EEHV1(NAP19) version used as the outgroup. The branch distance scale and some representative distance values are given. All six examples that have classic EEHV1B core chimeric domain (CD) features (see Fig 1) are marked with solid circle motifs. (TIFF) [file pone.0202438.s016.tiff]

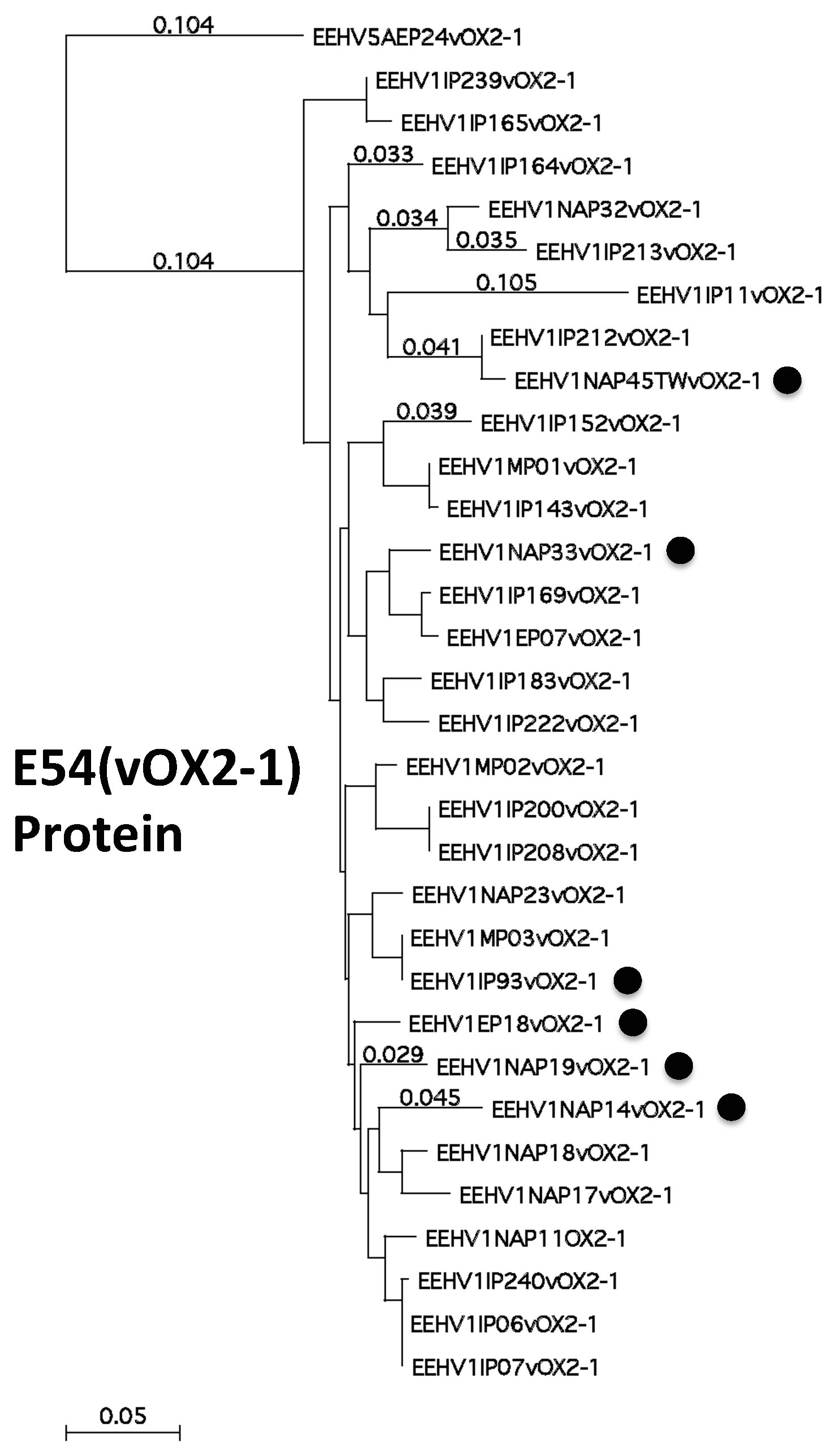

Supplement: S17 Fig — Bayesian linear phylogenetic tree generated from translated amino acid data in MEGA5 by the maximum likelihood method from the matching dataset as in the S7 and S14 Figs and with EEHV5A(EP24 = Vijay) used as the outgroup. The branch distance scale and some representative distance values are given. All six examples that have classic EEHV1B core chimeric domain (CD) features (see Fig 1) are marked with solid circle motifs. (TIFF) [file pone.0202438.s017.tiff]
